# Endothelial Cell-Specific Molecule 2 (ECSM2) Localizes to Cell-Cell Junctions and Modulates bFGF-Directed Cell Migration via the ERK-FAK Pathway

**DOI:** 10.1371/journal.pone.0021482

**Published:** 2011-06-24

**Authors:** Chunwei Shi, Jia Lu, Wen Wu, Fanxin Ma, Joseph Georges, Hanju Huang, James Balducci, Yongchang Chang, Yao Huang

**Affiliations:** 1 Department of Obstetrics and Gynecology, St. Joseph's Hospital and Medical Center, Phoenix, Arizona, United States of America; 2 Barrow Neurological Institute, St. Joseph's Hospital and Medical Center, Phoenix, Arizona, United States of America; 3 Department of Pathogen Biology, Tongji Medical College, Huazhong University of Science and Technology, Wuhan, China; 4 State Key Laboratory of Biotherapy, West China Hospital, College of Life Science, Sichuan University, Chengdu, China; Kings College London, United Kingdom

## Abstract

**Background:**

Despite its first discovery by *in silico* cloning of novel endothelial cell-specific genes a decade ago, the biological functions of endothelial cell-specific molecule 2 (ECSM2) have only recently begun to be understood. Limited data suggest its involvement in cell migration and apoptosis. However, the underlying signaling mechanisms and novel functions of ECSM2 remain to be explored.

**Methodology/Principal Findings:**

A rabbit anti-ECSM2 monoclonal antibody (RabMAb) was generated and used to characterize the endogenous ECSM2 protein. Immunoblotting, immunoprecipitation, deglycosylation, immunostaining and confocal microscopy validated that endogenous ECSM2 is a plasma membrane glycoprotein preferentially expressed in vascular endothelial cells (ECs). Expression patterns of heterologously expressed and endogenous ECSM2 identified that ECSM2 was particularly concentrated at cell-cell contacts. Cell aggregation and transwell assays showed that ECSM2 promoted cell-cell adhesion and attenuated basic fibroblast growth factor (bFGF)-driven EC migration. Gain or loss of function assays by overexpression or knockdown of ECSM2 in ECs demonstrated that ECSM2 modulated bFGF-directed EC motility via the FGF receptor (FGFR)-extracellular regulated kinase (ERK)-focal adhesion kinase (FAK) pathway. The counterbalance between FAK tyrosine phosphorylation (activation) and ERK-dependent serine phosphorylation of FAK was critically involved. A model of how ECSM2 signals to impact bFGF/FGFR-driven EC migration was proposed.

**Conclusions/Significance:**

ECSM2 is likely a novel EC junctional protein. It can promote cell-cell adhesion and inhibit bFGF-mediated cell migration. Mechanistically, ECSM2 attenuates EC motility through the FGFR-ERK-FAK pathway. The findings suggest that ECSM2 could be a key player in coordinating receptor tyrosine kinase (RTK)-, integrin-, and EC junctional component-mediated signaling and may have important implications in disorders related to endothelial dysfunction and impaired EC junction signaling.

## Introduction

Angiogenesis is not only essential for normal organ growth, development and wound healing, but also an important determinant for many diseases such as cancer, atherosclerosis, diabetic retinopathies, and rheumatoid arthritis [1], [2]. Endothelial cells (ECs) that line the lumina of blood vessels are important players in blood vessel formation, and directed EC migration is a key component of the angiogenic process. Accordingly, there has been a long-standing interest in identifying genes specifically or preferentially expressed in ECs and understanding their biological functions. This may lead to the discovery of new pathways and molecular targets with therapeutic potentials. Endothelial cell-specific molecule 2 (*ECSM2*) [3], [4], also known as endothelial cell-specific chemotaxis receptor (*ECSCR*) [5] and apoptosis regulator through modulating cIAP expression (*ARIA*) [6], was initially identified a decade ago by *in silico* cloning of novel EC-specific genes [7]. Although human ECSM2 was predicted to encode a hypothetical protein with a suggested role in cell adhesion based on its putative amino acid profile [7], its biological and cellular functions have only recently begun to be understood.

We and others have independently demonstrated that a family of evolutionarily conserved *ECSM2* genes from a variety of species is preferentially expressed in ECs and vasculature [3], [4], [5], [6]. These studies also suggest that ECSM2 is involved in cell migration, angiogenesis and apoptosis albeit some of the results are controversial [8]. The effects of ECSM2 on cell migration could be related to actin remodeling [3], [4] via crosstalk with receptor tyrosine kinases (RTKs), such as epidermal growth factor receptor (EGFR) [3] and vascular endothelial growth factor receptor (VEGFR) [5]. ECSM2 is emerging as a promising therapeutic target due to its endothelial specificity and potential roles in EC migration and apoptosis [8]. However, novel functions of ECSM2 and its signaling mechanisms remain to be elucidated, which are primary goals of the present study.

Among numerous growth factors that have been implicated in angiogenesis and vascular remodeling, basic fibroblast growth factor (bFGF) is a potent angiogenic inducer that can stimulate EC migration and proliferation via interaction with its specific receptor FGFR, a member of the RTK superfamily [9], [10], [11], [12]. In this study, we focus on the impact of ECSM2 on bFGF/FGFR actions in ECs. Using multiple experimental approaches, we provide strong evidence suggesting that ECSM2 is an EC junctional protein and promotes cell-cell adhesion. We further demonstrate that ECSM2 can inhibit bFGF-driven cell motility via the extracellular regulated kinase (ERK)-focal adhesion kinase (FAK) pathway. Finally, we provide a model of how ECSM2 contributes to the regulation of EC migration. Our novel findings suggest that ECSM2 could be a key player in coordinating RTK-, integrin-, and EC junctional component-mediated signaling. Given the importance of RTK, adhesion and junction signaling, the current work also lays a foundation for future studies of more detailed roles of ECSM2 within the signaling network of ECs.

## Results

### Generation of anti-ECSM2 monoclonal antibody and characterization of endogenous ECSM2

**Figure 1:**
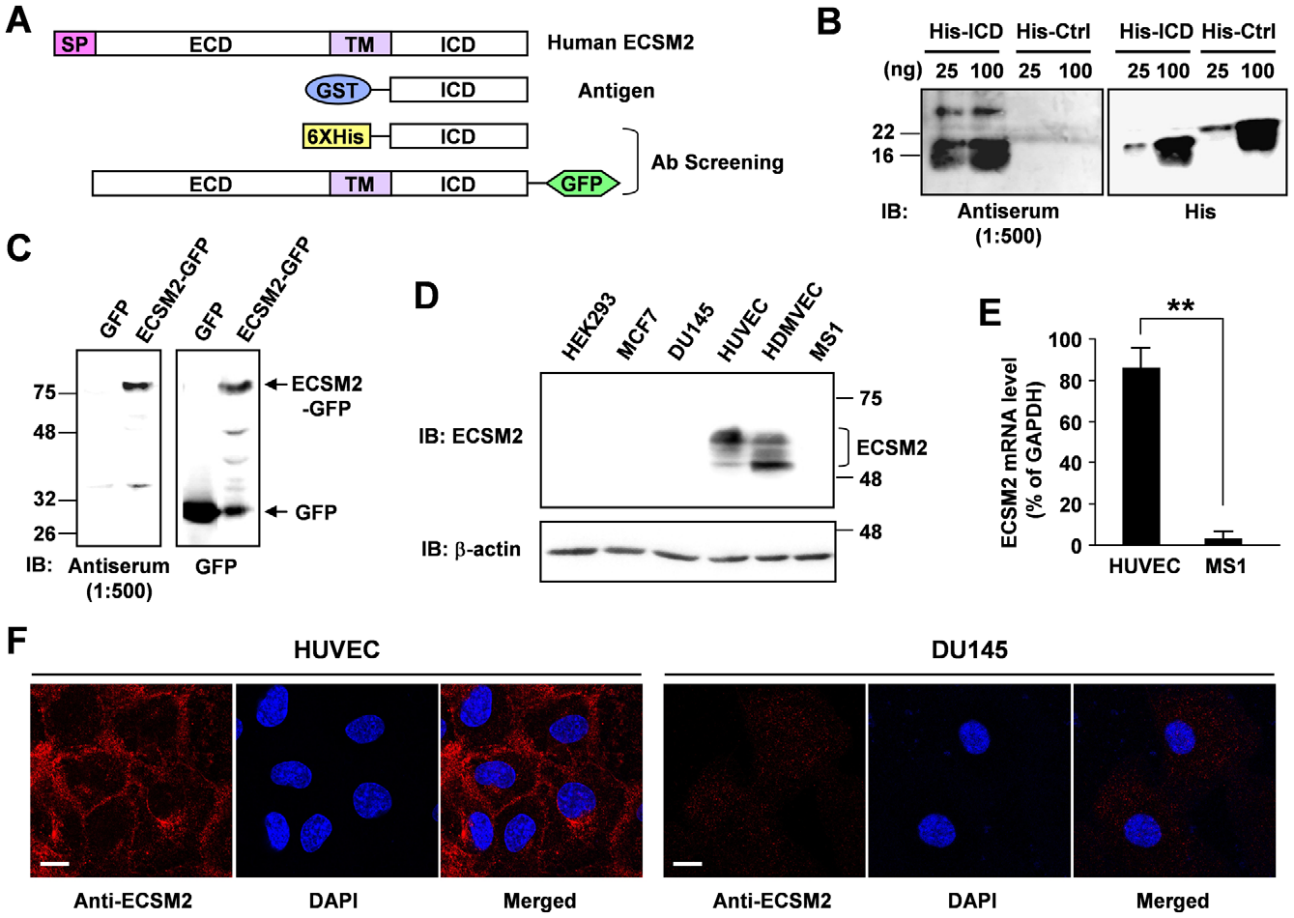
Generation of rabbit anti-ECSM2 monoclonal antibody (RabMAb) and characterization of endogenous ECSM2. (A) Diagram of human ECSM2 constructs used in generation and screening of anti-ECSM2 antibodies. SP, signal peptide; TM, transmembrane domain; ECD, extracellular domain; ICD, intracellular domain. (B) Anti-ECSM2 antiserum specifically detects His-ICD. Purified GST-ICD proteins were used to immunize the rabbit to generate polyclonal antibody (pAb) and monoclonal antibody (RabMAb) sequentially as detailed in *Methods*. Antiserum (bleed containing pAb) was collected and used for immunoblotting. His-Ctrl: His-tagged non-related protein (control). (C) Anti-ECSM2 pAb recognizes ECSM2-GFP but not GFP. Protein extracts from the HEK293 cells overexpressing human ECSM2-GFP or GFP alone were used for immunoblotting. (D) Anti-ECSM2 RabMAb specifically detects endogenous ECSM2 in human ECs. Protein extracts from human EC lines (HUVEC and HDMVEC), mouse endothelial MS1 cells, and non-EC lines (MCF-7, DU145 and HEK293) were analyzed by immunoblotting with anti-ECSM2 RabMAb subclone 71-1 (hybridoma supernatant) or anti-β-actin (loading control). (E) Quantitative RT-PCR analysis measuring the ECSM2 mRNA levels in HUVEC and MS1 cells, normalized to human and mouse GAPDH, respectively. Data are mean±SEM (n = 6). **, *P*<0.01. (F) Plasma membrane localization of endogenous ECSM2. HUVEC was costained with anti-ECSM2 RabMAb (subclone 71-1) (*red*) and DAPI (*blue*), and visualized by confocal microscopy. Human prostate cancer DU145 cells were used as a negative control for anti-ECSM2. Scale bar, 20 µm.

We and others have recently demonstrated that the ECSM2 gene is preferentially expressed in vascular ECs largely by means of quantitative RT-PCR and in situ hybridization [3], [4]. Bioinformatics analysis and heterologous expression of GFP-, myc-, or FLAG-tagged ECSM2 proteins in several mammalian cell systems further suggested that ECSM2 is a cell membrane protein consisting of an N-terminal extracellular domain (ECD), a single transmembrane domain (TM), and a small, highly conserved C-terminal intracellular domain (ICD) [3], [4], [6]. To study the endogenous ECSM2 protein, here we generated rabbit anti-ECSM2 monoclonal antibodies (RabMAb) using a GST fusion protein containing the entire ICD of human ECSM2 as the immunogen (Figure 1). One hybridoma subclone (RabMAb 71-1) specifically detected the endogenous ECSM2 proteins in human EC lines (HUVEC and HDMVEC) by immunoblotting (Figure 1D). The appearance of diffuse, multiple bands on SDS-PAGE (50–60 kDa, significantly larger than the predicted molecular mass of 25–30 kDa) could be due to some posttranslational modifications such as glycosylation. Notably, this RabMAb subclone can hardly detect the ECSM2 in mouse endothelial MS1 cells (Figure 1D) although quantitative RT-PCR revealed the presence of ECSM2 mRNA in MS1 (Figure 1E). However, its mRNA level in MS1 cells was only 3.8±2.2% of that in HUVEC when normalized to GAPDH (Figure 1E). This indicated that the endogenous ECSM2 level was indeed low in MS1 cells, making it an appealing system to investigate the cellular functions of ECSM2 in ECs by overexpression (see below). In addition, the cell membrane localization of endogenous ECSM2 in HUVEC can be detected by immunostaining with this RabMAb and confocal microscopy (Figure 1F), which confirmed the previous findings of heterologously expressed tagged ECSM2 proteins [3], [4], [6].

**Figure 2:**
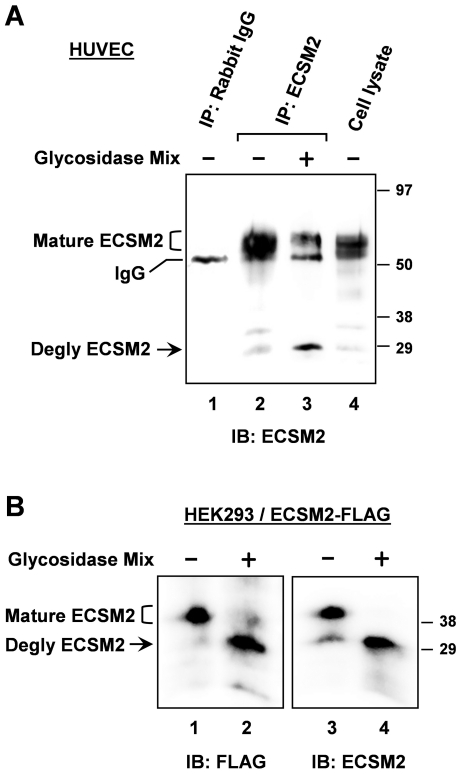
Characterization of ECSM2 proteins by enzymatic deglycosylation. (A) Glycosylation of endogenous ECSM2. HUVEC lysates were immunoprecipitated with anti-ECSM2 RabMAb (lanes 2 and 3) or rabbit IgG as a control (lane 1). Samples were treated with (+) or without (-) glycosidase mix, as detailed in *Methods*, resolved by SDS-PAGE, and immunoblotted with anti-ECSM2 RabMAb. Positions of glycosylated (mature) ECSM2, deglycosylated ECSM2, and IgG are indicated. (B) Glycosylation of ECSM2-FLAG. HEK293 cells stably expressing mouse ECSM2-FLAG were lysed and cell lysates were directly subjected to enzymatic deglycosylation reactions as described in *Methods*. Samples were analyzed by immunoblotting with anti-FLAG M2 mAb (lanes 1 and 2) and anti-ECSM2 RabMAb (lanes 3 and 4), respectively. Positions of glycosylated (mature) ECSM2-FLAG, and deglycosylated ECSM2-FLAG are indicated.

It has been hypothesized that ECSM2 is a heavily glycosylated protein based on at least two facts. First, there are several conserved *N*- and *O*-linked glycosylation sites in the extracellular domain of ECSM2 proteins across species. Second, the size of heterologously expressed ECSM2 protein is nearly twice of predicted molecular mass from the amino acid sequences [3], [4], [6] (also see Figure 1D for endogenous ECSM2). For many glycoproteins, the particular patterns of glycosylation dictate specific alterations in their migration on SDS-PAGE as demonstrated previously [13]. Thus, we performed enzymatic deglycosylation experiments to test whether the difference between the actual and predicted sizes of ECSM2 was related to glycosylation (Figure 2). We first enriched the ECSM2 protein from total cell lysates of HUVEC by immunoprecipitation (IP) with anti-ECSM2 RabMAb and then treated it with a mixture of glycosidases to remove both *N*- and *O*-glycans under denaturing conditions. As shown in Figure 2A, after resolved by SDS-PAGE and immunoblotted with anti-ECSM2, the majority of precipitated proteins migrated much more rapidly upon deglycosylation, changing from 50–60 kDa to ∼30 kDa (Figure 2A, lane 3 vs. 2). Notably, however, there remained some fractions with molecular mass of 50–60 kDa in the glycosidase-treated sample (Figure 2A, lane 3), suggesting that other mechanisms may contribute to the retardation of ECSM2 migration on SDS-PAGE in this cell context (see Discussion). To corroborate the finding of glycosylation, we performed non-IP-based deglycosylation assays using HEK293 cells overexpressing ECSM2-FLAG (Figure 2B). A nearly complete downshift of ECSM2-FLAG migration (from ∼40 kDa to ∼30 kDa) on SDS-PAGE was revealed by immunoblotting with both anti-FLAG and anti-ECSM2 (Figure 2B, lane 2 vs. 1, lane 4 vs. 3), indicating that ECSM2-FLAG also undergoes glycosylation. In addition, we noted the difference in molecular mass of endogenous, mature ECSM2 in HUVEC (50–60 kDa) versus heterologously expressed, mature ECSM2-FLAG (∼40 kDa) in HEK293 cells (see Discussion).

### Identification of ECSM2 as a novel cell-cell junctional protein

We have previously examined the impact of ECSM2 on EGF-directed cell migration by coexpression of ECSM2-GFP and EGFR in a non-EC cell line, HEK293 [3]. Here we sought to further explore novel ECSM2 functions and signaling mechanisms underlying its effects on cell motility in a more physiologically relevant endothelial system. As endogenous ECSM2 expression is low in MS1 cells (Figure 1D and 1E), we established the MS1 cells stably overexpressing ECSM2-GFP (referred to as MS1/ECSM2-GFP) or GFP alone (referred to as MS1/GFP). As expected, ECSM2-GFP appeared highly glycosylated with a much larger molecular mass than predicted (∼75 versus ∼50 kDa) (Figure S1A) and was localized to the cell membrane (Figure S1B). As we previously reported for MS1 transient transfectants [3], the filopodia-like structures were evident in the MS/ECSM2-GFP stable cell line when cell density was low (Figure S1B, *arrowheads*). Surprisingly, when cells reached a high density, we observed significantly enhanced cell-cell junctional localization of ECSM2-GFP (Figure S1C, *arrows*). Similar patterns were also detected in HEK293 cells overexpressing ECSM2 tagged with either GFP or FLAG (Figure S1D).

**Figure 3:**
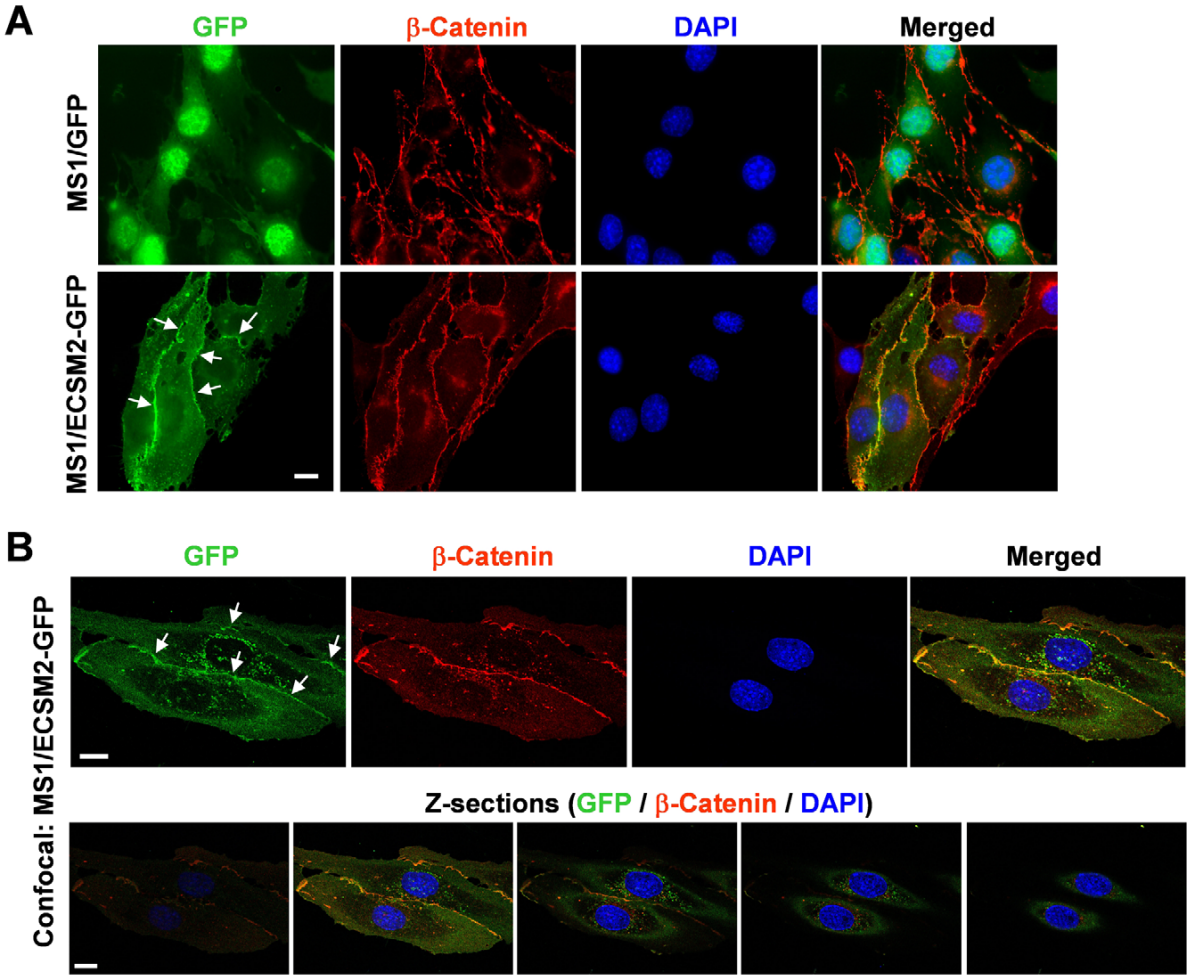
Colocalization of ECSM2-GFP with β-catenin. (A) Colocalization of ECSM2-GFP with β-catenin revealed by standard fluorescent microscopy. MS1 cells expressing GFP (control) or ECSM2-GFP were costained with anti-β-catenin antibody and DAPI. Merged images of GFP (*green*), β-catenin (*red*), and DAPI (*blue*) staining are shown. Scale bar, 20 µm. (B) Colocalization of ECSM2-GFP with β-catenin confirmed by confocal microscopy. Z-sections of a representative image are also shown as merged results of GFP (*green*), β-catenin (*red*), and DAPI (*blue*). Scale bar, 20 µm.

**Figure 4:**
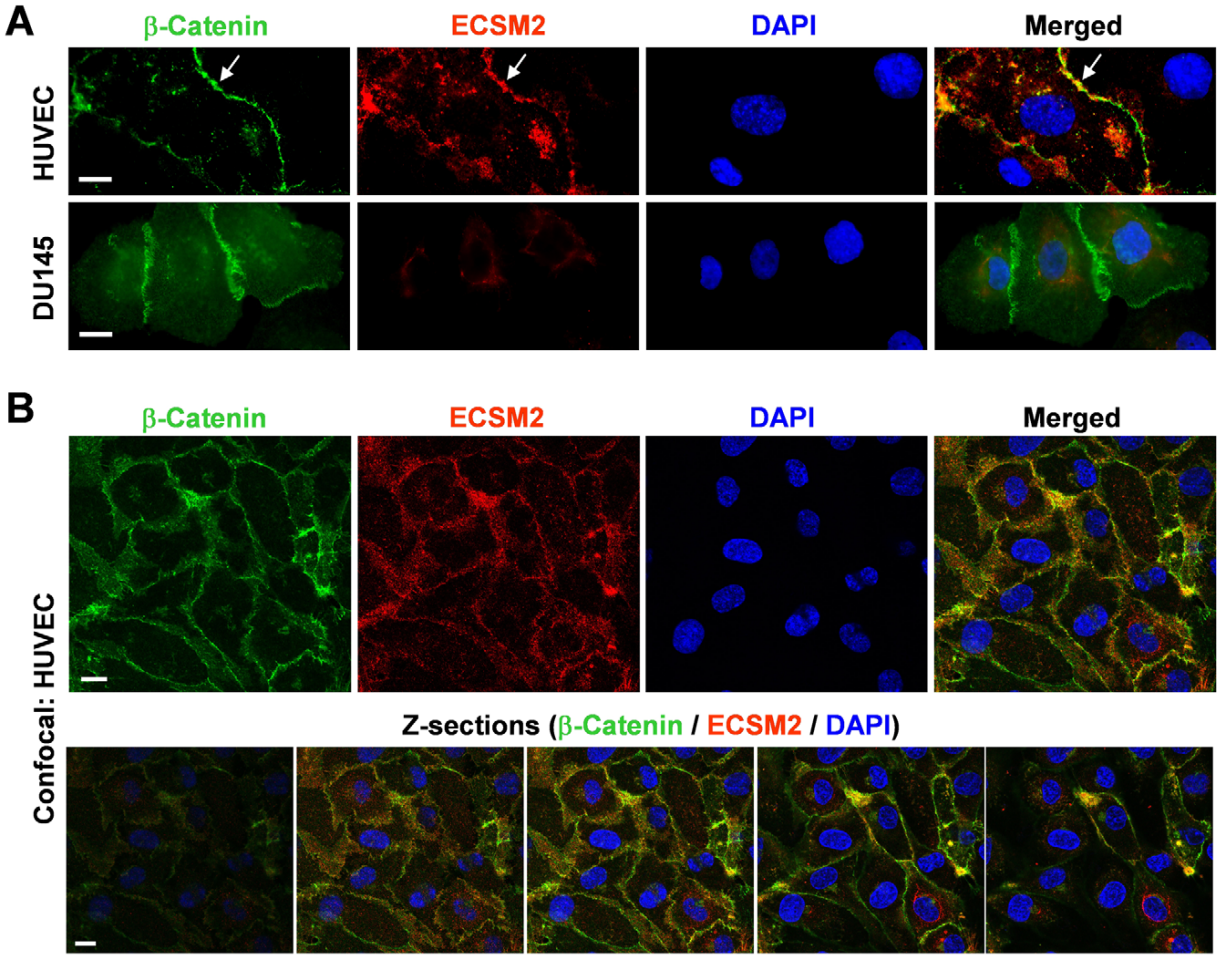
Endogenous ECSM2 also localizes to cell-cell junctions. (A) Colocalization of endogenous ECSM2 with β-catenin revealed by standard fluorescent microscopy. HUVEC or DU145 (control) cells were costained with anti-ECSM2 RabMAb (subclone 71-1), anti-β-catenin, and DAPI. Merged images of β-catenin (*green*), ECSM2 (*red*), and DAPI (*blue*) staining are shown. Overlapping distribution of endogenous ECSM2 and β-catenin in HUVEC is indicated by *arrows*. Scale bar, 20 µm. (B) Colocalization of endogenous ECSM2 with β-catenin in HUVEC, confirmed by confocal microscopy. Z-sections of a representative image are also shown as merged results of β-catenin (*green*), ECSM2 (*red*), and DAPI (*blue*). Scale bar, 20 µm.

β-catenin, a well-acknowledged component of the cadherin-catenin complex constituting adherens junctions [14], [15], has been successfully utilized as a cell-cell junctional marker in our previous study [16]. We thus immunostained the MS1/ECSM2-GFP cells with an anti-β-catenin antibody. The results indicated that ECSM2-GFP was markedly colocalized with β-catenin (Figure 3A). We further performed confocal microscopy and optical sectioning along the Z axis (Z-sections). The results confirmed that ECSM2 and β-catenin were indeed mainly colocalized at cell-cell junctions (Figure 3B). In contrast, ECSM2-GFP did not localize to focal adhesions (FAs) where abundant FAK (a marker of FAs [16], [17]) was detected (Figure S2). Similarly, we also examined the subcellular distribution of endogenous ECSM2 in HUVEC by coimmunostaining with anti-ECSM2 RabMAb and anti-β-catenin, in which a non-EC human cell line DU145 was used as a control for anti-ECSM2 RabMAb (Figure 4A). Both standard fluorescent microscopy (Figure 4A) and confocal microscopy including Z-sectioning (Figure 4B) identified the overlapping distributions of ECSM2 and β-catenin in HUVEC. Notably, unlike the β-catenin staining (seen as a sharp, more defined belt along the cell contact area in general), the distribution of ECSM2 in HUVEC appeared to be rather less focused, although they did extensively overlap (Figure 4). This finding may reflect the molecular diversity and complexity of cell adhesions in ECs (see Discussion).

### ECSM2 promotes cell-cell adhesion and inhibits bFGF-directed cell migration

**Figure 5:**
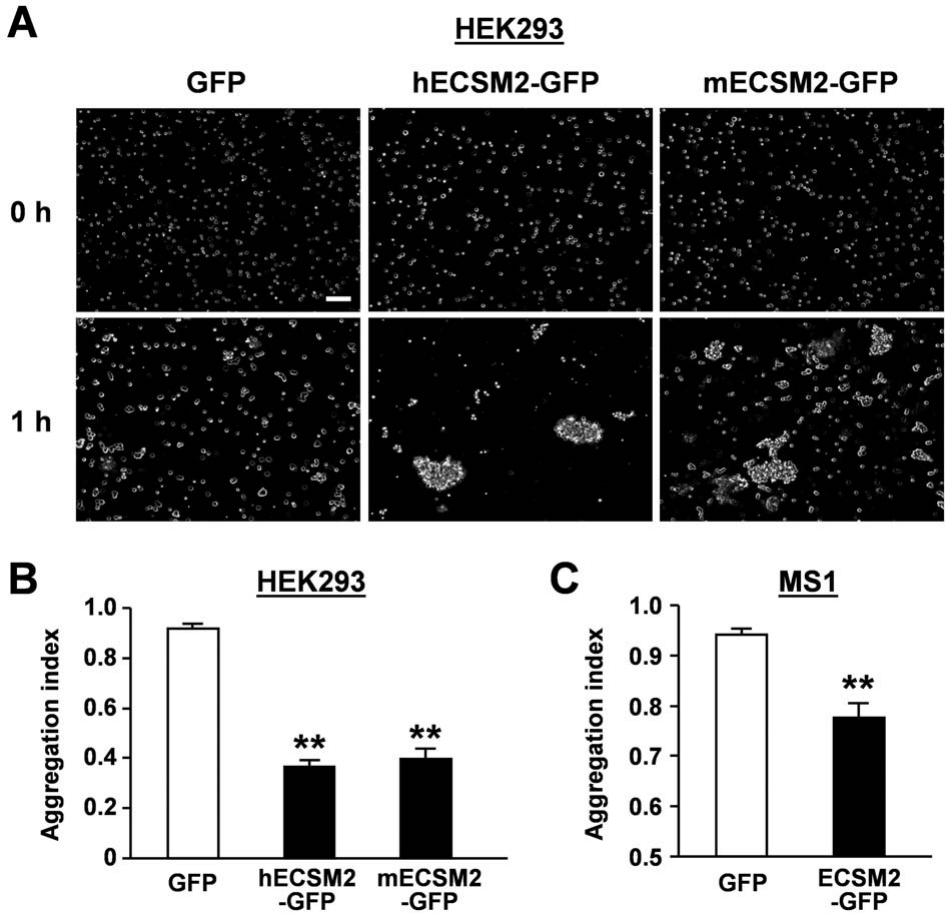
Overexpression of ECSM2 promotes cell aggregation. (A) HEK293 cells stably expressing GFP (control), human (h) or mouse (m) ECSM2-GFP were used for cell aggregation assays, as described in *Methods*. Representative images captured by an inverted phase contrast microscope (magnification: ×10) are shown. Scale bar, 200 µm. (B and C) The aggregation indexes for HEK293 stably expressing GFP, human (h) or mouse (m) ECSM2-GFP (B), and MS1 stably expressing GFP or human ECSM2-GFP (C) were calculated and plotted. Data are mean±SEM (n = 8). **, *P*<0.01.

The prominent localization of ECSM2 to intercellular junctions prompted us to investigate its potential roles in cell-cell adhesion and cell motility, two related cellular processes essential for many key endothelial functions. We performed cell aggregation assays in both HEK293 and MS1 cell systems. In the representative experiment using HEK293 cells stably overexpressing GFP, human and mouse ECSM2-GFP, respectively, shown in Figure 5A, large cell-cell aggregates were seen in the cells expressing ECSM2-GFP but not in those only expressing GFP. A total of approximately 1,000 cells were counted under each condition for both HEK293 and MS1 cell systems and the aggregation indexes were calculated. As displayed in Figure 5B and 5C, forced expression of ECSM2-GFP in both HEK293 and MS1 cells significantly promoted cell-cell adhesion when compared to the GFP control (*P*<0.01).

**Figure 6:**
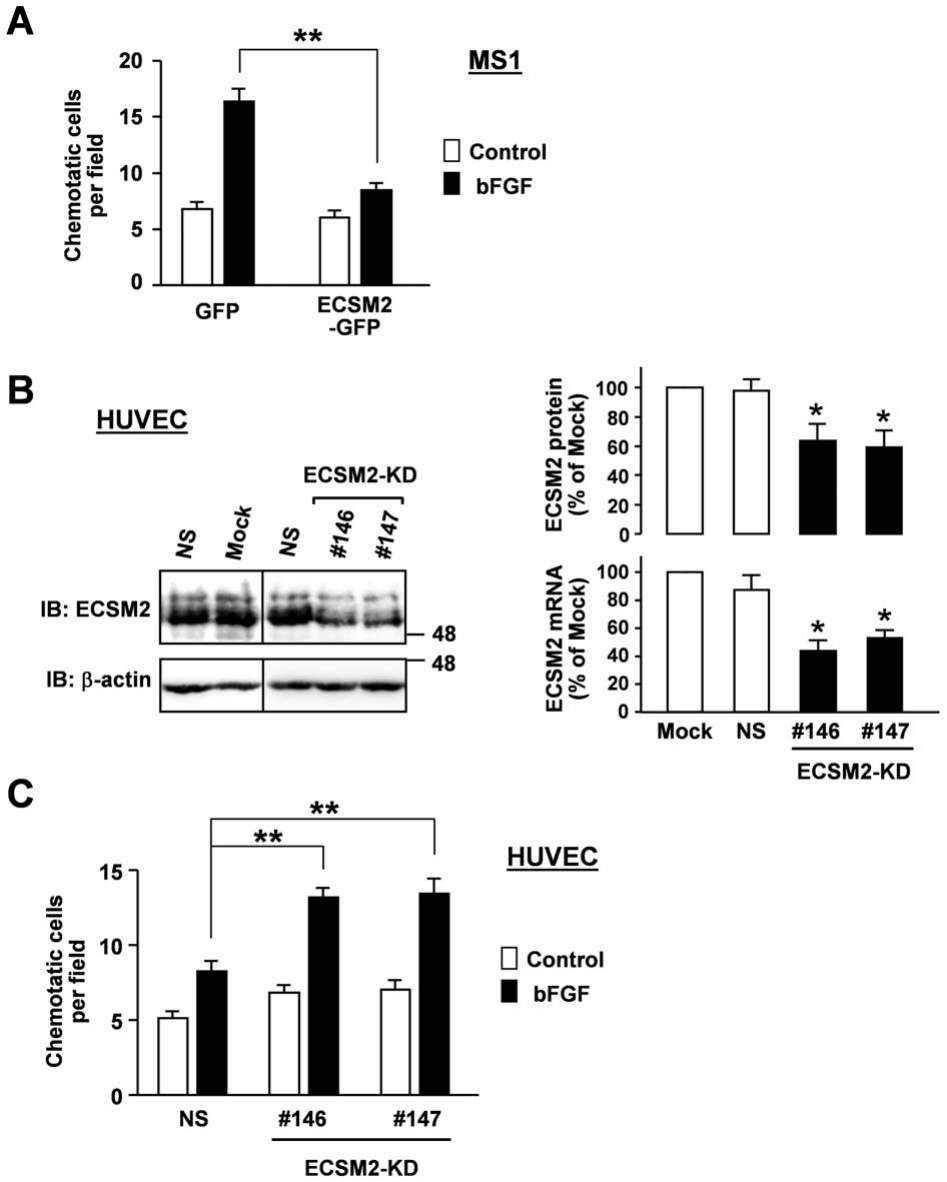
Effects of ECSM2 on bFGF-directed cell migration. (A) Overexpression of ECSM2-GFP attenuates bFGF-induced cell motility measured by transwell assays. MS1 cells expressing GFP or ECSM2-GFP were allowed to migrate across the membrane of the transwell insert in response to bFGF (10 ng/ml) in serum-free media for 5 h. The number of migrated cells per imaging field for each condition was counted. Data are mean±SEM (n = 12). **, *P*<0.01. (B) Knockdown of endogenous ECSM2 in HUVEC. HUVECs were transfected with ECSM2 siRNAs (sequence no. 146 or 147) or nonspecific (NS) siRNA. Total RNAs and proteins were extracted from the cells 48 and 72 h post-transfection, respectively, and used for quantitative PCR and immunoblotting analyses. Statistical results of three independent experiments are shown. Data are mean±SEM. *, *P*<0.05. (C) Knockdown of ECSM2 enhances bFGF-induced cell migration. HUVECs were transfected with nonspecific or ECSM2 siRNAs (sequence no. 146 or 147) for 72 h and used for transwell assays. The cells were allowed to migrate in response to bFGF (10 ng/ml) in serum-free and ECGS-free media for 5 h. The number of migrated cells per imaging field for each condition was counted. Data are mean±SEM (n = 12). **, *P*<0.01.

We have previously shown that forced expression of ECSM2-GFP in HEK293/EGFR cells (constitutively expressing EGFR) inhibits the EGF-induced cell migration [3]. Here we sought to investigate the effects of ECSM2 on bFGF/FGFR-driven motility in ECs, given that bFGF has been considered as an angiogenic factor and bFGF/FGFR signaling plays an important role in vascular development and EC functions including migration [10], [12]. Since MS1 and HUVEC cells are highly responsive to bFGF, reflected in bFGF-induced robust ERK activation [18] (Figure S3), we assessed bFGF-directed EC migration by transwell assays. The results indicated that overexpression of ECSM-GFP in MS1 cells significantly attenuated bFGF-induced motility (Figure 6A).

As an alternative approach, we performed siRNA knockdown experiments. Transfection of ECSM2 siRNA, but not nonspecific (NS) control siRNA, into HUVEC significantly reduced the ECSM2 expression at both mRNA and protein levels (Figure 6B). Importantly, the immunoblotting results (Figure 6B, *left panel*) further confirmed the specificity of our anti-ECSM2 RabMAb. In contrast to the effects on cell motility by overexpression of ECSM2-GFP in MS1 cells, knockdown of ECSM2 (ECSM2-KD) in HUVEC significantly enhanced the bFGF-induced cell migration (Figure 6C). Taken together, our data demonstrate that ECSM2 is likely a previously unappreciated intercellular junction protein, promotes cell-cell adhesion, and inhibits bFGF-directed cell migration.

### Signaling mechanism underlying the effect of ECSM2 on bFGF-driven cell migration

**Figure 7:**
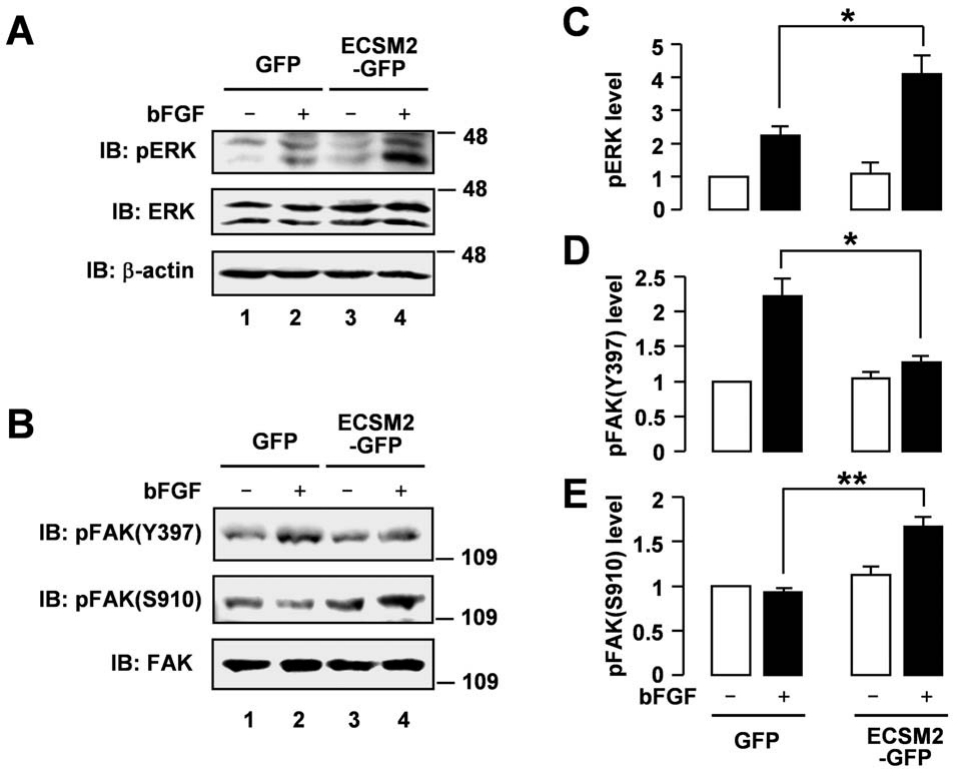
Overexpression of ECSM2 affects bFGF-induced ERK and FAK signaling. (A and B) Serum-starved MS1 cells expressing GFP or ECSM2-GFP were stimulated with vehicle (-) or bFGF (10 ng/ml) for 15 min. Protein extracts were analyzed by immunoblotting with anti-pERK, anti-total ERK, anti-β-actin, anti-pFAK(Y397), anti-pFAK(S910), or anti-total FAK, as indicated. (C–E) Statistical analysis (densitometry) of pooled data of pERK, pFAK(Y397), and pFAK(S910) from four independent experiments. Data are mean±SEM. *, *P*<0.05; **, *P*<0.01.

We further probed the signaling mechanism underlying the effect of ECSM2 on cell motility. As mentioned above, bFGF caused robust ERK activation in both MS1 and HUVEC cells (Figure S3). Here we assessed the bFGF-elicited signaling in the ECSM2 overexpression system. Interestingly, overexpression of ECSM2-GFP in MS1 cells enhanced bFGF-induced ERK activation compared to the GFP control (Figure 7A, *upper panel*, lane 4 vs. 2). ERK has been implicated in the motility of numerous cell types stimulated by growth factors including VEGF, FGF, EGF, platelet-derived growth factor (PDGF), and insulin, which involves phosphorylation of FAK [19], [20]. Surprisingly, overexpression of ECSM2 inhibited bFGF-mediated FAK phosphorylation at Tyr397, the major autophosphorylation site [21], [22] (Figure 7B, *upper panel*, lane 4 vs. 2). Similar results were obtained for phosphorylation at Tyr576 and Tyr577 sites (Figure S4), which is required for FAK maximal activation [23]. The diminished FAK activation upon ECSM2 overexpression was in agreement with reduced motility in these cells (Figure 6A).

In addition to tyrosine phosphorylation, FAK can be phosphorylated at several serine residues including Ser910 in response to a variety of stimuli, and this requires ERK activation [20], [24]. Thus, we examined whether the reduced pFAK(Y397) level was related to enhanced pFAK(S910) (presumably by pERKs) in our cell system. Indeed, an elevated pFAK(Ser910) level (bFGF-induced) was detected in MS1/ECSM2-GFP cells (Figure 7B, *middle panel*, lane 4 vs. 2). Taken together, the pERK level displayed a positive and negative relationship with pFAK(S910) and pFAK(Y397) levels, respectively (Figure 7C–E).

**Figure 8:**
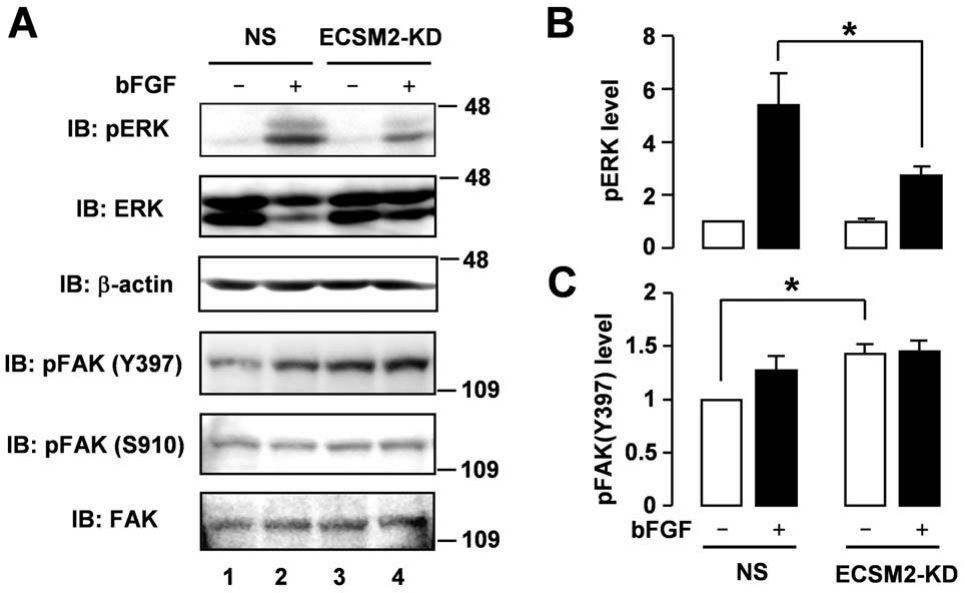
The effects of ECSM2 knockdown on bFGF-induced ERK and FAK signaling. (A) HUVECs were transfected with nonspecific (NS) or ECSM2 siRNAs for 72 h, starved for 8 h, and then stimulated with vehicle (-) or bFGF (10 ng/ml) for 15 min. Protein extracts were analyzed by immunoblotting with anti-pERK, anti-total ERK, anti-β-actin, anti-pFAK(Y397), anti-pFAK(S910), or anti-total FAK, as indicated. (B and C) Statistical analysis of pooled data of pERK and pFAK(Y397) from three independent experiments. Data are mean±SEM. *, *P*<0.05.

We also evaluated the signaling profiles in the HUVEC system where endogenous ECSM2 was knocked down by RNA interference (Figure 6B). ECSM2-KD significantly inhibited bFGF-induced ERK activation (Figure 8A and 8B) but enhanced pFAK(Y397) under the basal condition (Figure 8A and 8C). In contrast, we did not detect an apparent difference in pFAK(S910) levels between the NS control and ECSM2-KD cells (Figure 8A; Statistical analysis of pFAK(S910) can be found in Figure S5). Interpretations of the interrelationships among pERK, pFAK(S910) and pFAK(Y397) in the cases of ECSM2 overexpression and knockdown using our model are further discussed below (see Discussion). Collectively, our novel findings suggest that FAK can be an important effector in the ECSM2-regulated migratory pathway in response to bFGF, although ECSM2 seems to be an intercellular junction protein (Figures 3, 4, 5) and absent at FAs (Figure S2).

### ERK resides upstream of FAK in the ECSM2-regulated migratory pathway

**Figure 9:**
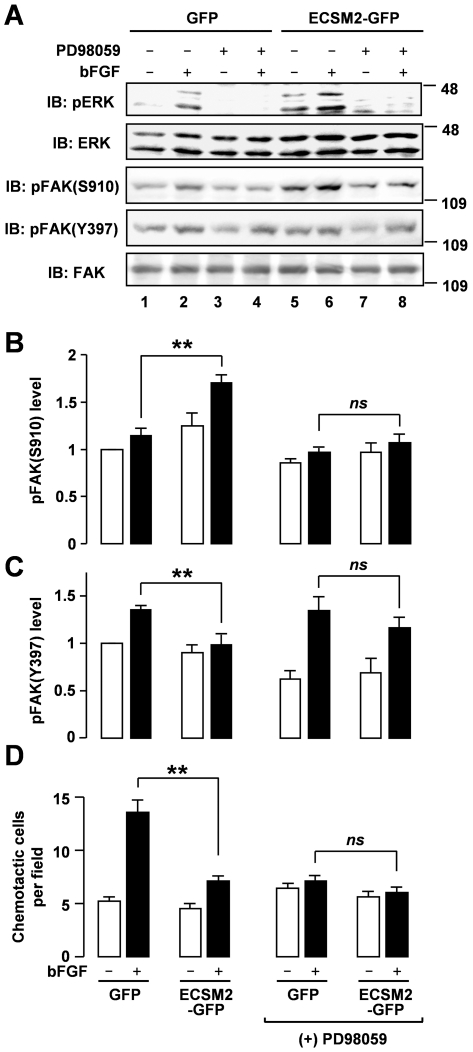
ERK resides upstream of FAK in ECSM2-regulated FGFR-ERK-FAK pathway. (A) Blockade of ERK activation eliminates the upregulation of pFAK(Y397) and the downregulation of pFAK(S910) (bFGF-induced) in MS1 cells expressing ECSM2-GFP versus GFP control cells. Serum-starved cells were pretreated with DMSO (control) or PD98059 (50 µM) for 1 h prior to stimulation with vehicle (-) or bFGF (10 ng/ml) for 15 min. Protein extracts were analyzed by immunoblotting with anti-pERK, anti-total ERK, anti-pFAK(S910), anti-pFAK(Y397), or anti-total FAK, as indicated. (B and C) Statistical analysis (densitometry) of pooled data of pFAK(S910) and pFAK(Y397) from three independent experiments. Data are mean±SEM. **, *P*<0.01; *ns*, not statistically significant. (D) Effect of inhibition of ERK activity on bFGF-induced cell migration. Serum-starved MS1 cells stably expressing GFP or ECSM2-GFP were pretreated with DMSO (control) or PD98059 (50 µM) for 1 h prior to transwell assays, as detailed in *Methods*. The cells were allowed to migrate across the membrane of the transwell insert in response to bFGF (10 ng/ml) in serum-free media for 5 h. The number of migrated cells per imaging field for each condition was counted. Data are mean±SEM (n = 12). **, *P*<0.01; *ns*, not statistically significant.

Previous studies have suggested a complicated relationship between ERK and FAK in cell migration [19]. To test whether ERK resides upstream or downstream of FAK within the ECSM2-regulated signaling cascades, we employed PD98059, a specific pharmacological inhibitor of MEK1 (the upstream kinase of ERKs), to block ERK activation [25], [26], [27], and then assessed FAK phosphorylation. As expected, PD98059 inhibited bFGF-induced ERK activation in both MS1/GFP and MS1/ECSM2-GFP cells (Figure 9A, *upper panel*). In the absence of PD98059, overexpression of ECSM2-GFP led to upregulation of bFGF-induced pFAK(S910) (*pFAK(S910) panel* in Figure 9A, and Figure 9B) and downregulation of bFGF-induced pFAK(Y397) (*pFAK(Y397) panel* in Figure 9A, and Figure 9C), as already observed (Figure 7). Notably, however, PD98059 treatment abolished the difference of bFGF-induced pFAK(S910) levels between MS1/GFP and MS1/ECSM2-GFP cells (Figure 9B). Interestingly, the drug also eliminated the difference of bFGF-induced pFAK(Y397) between the two cell lines (Figure 9C).

We further assessed bFGF-driven migration of MS1/GFP and MS1/ECSM2-GFP cells in the presence or absence of PD98059 by transwell assays (Figure 9D). As previously shown in Figure 6A, without PD98059 treatment, overexpression of ECSM2-GFP significantly reduced bFGF-directed motility compared to the GFP control (*P*<0.01) (Figure 9D). In contrast, inhibition of the MEK/ERK pathway by PD98059 abrogated the difference of bFGF-induced motility between MS1/GFP and MS1/ECSM2-GFP cells (Figure 9D). Meanwhile, we noted that PD98059 also reduced bFGF-directed migration in MS1/GFP cells (Figure 9D). This is not surprising as several previous studies have demonstrated that blockade of ERK signaling attenuates cell migration induced by growth factors such as VEGF and bFGF [28], [29], [30], [31] and the Ras/Raf/MEK/ERK cascade is an essential effector required for most RTK function [32]. Taken together, we conclude that ERK resides upstream of FAK within the ECSM2-regulated migratory pathway in this cell context.

## Discussion

Experimental validation of ECSM2 as an endothelial specific protein and recent findings of its involvement in cell migration, angiogenesis, and apoptosis [3], [4], [5], [6] provide an exciting new opportunity to exploit the signaling network behind, to acquire a clear molecular and subcellular picture of ECSM2 within the network, and to study endothelial function/dysfunction related to this important, new molecule. To this end, by employing multiple approaches in the current study, we demonstrate that, being a plasma membrane protein, ECSM2 is concentrated at cell-cell contacts and promotes cell aggregation, suggesting that it is most likely a novel EC junction component. Importantly, we define that ECSM2 attenuates bFGF-mediated cell migration through the FGFR-ERK-FAK pathway.

Previous studies using RT-PCR, Northern blotting, in situ hybridization, bioinformatics analysis, and ectopic expression of tagged ECSM2 proteins have suggested that ECSM2 is a heavily glycosylated, cell plasma membrane protein preferentially expressed in vascular ECs [3], [4], [6]. In this study, we successfully generated a rabbit anti-ECSM2 monoclonal antibody (RabMAb). Using this RabMAb combined with immunoblotting, immunoprecipitation, enzymatic deglycosylation, immunostaining, and confocal microscopy, we have experimentally validated the endogenous characteristics of ECSM2. In particular, we have demonstrated that both endogenous and tagged ECSM2 can undergo glycosylation in cells. However, we note that the molecular mass of glycosylated ECSM2-FLAG overexpressed in HEK293 cells was approximately 10–20 kDa smaller than that of the endogenous mature ECSM2 in HUVEC (Figure 2). This may reflect the nature of the glycosylation process within the two different cell systems and/or incomplete glycosylation for constitutively overexpressed proteins. We also note that some mature ECSM2 proteins in HUVEC did not downshift to the deglycosylated form on SDS-PAGE after enzymatic deglycosylation. Although the glycosidase mix used includes all common enzymes (see Methods for details) for removal of almost all *N*-linked, all simple core 1 and core 3 *O*-linked, and certain long chain *O*-linked glycans, we can not rule out the possibility that Endoglycosidase H and additional exoglycosidases may be needed to release otherwise resistant sugars from ECSM2. The other possibility is that posttranslational modifications in addition to glycosylation (e.g. ubiquitination) could be involved since a role of ECSM2 in modulation of protein proteasomal degradation was previously reported [6].

The most exciting, intriguing finding from the present study is the concentrated distribution of ECSM2 at cell-cell contacts. Our data of enhanced homophilic cell-cell interactions by ECSM2 overexpression in both HEK293 and MS1 cells highly support that ECSM2 is most likely a previously unappreciated EC junctional protein. As a cell adhesion molecule (CAM), ECSM2 does not belong to any of the known CAM superfamilies including integrins, cadherins, immunoglobulins (IgSF), and selectins [33], [34], based on their primary sequence/structure comparison (data not shown). Endothelial cell-cell junctions are specialized membrane domains that not only maintain the integrity of endothelium, but also play critical roles in cell-cell communications and vascular permeability [35]. Similar to epithelial cells, ECs have two major types of junctions, namely adherens junctions (AJ) and tight junctions (TJ). However, the endothelial junction architecture is less defined and AJ and TJ are often intermingled, whereas epithelial junctions are better organized with TJ localizing apical to AJ [35]. The finding that both heterologously expressed and endogenous ECSM2 proteins were largely colocalized with β-catenin (an AJ component) at cell-cell contact areas, which was confirmed by confocal microscopy and Z-sectioning, implies that ECSM2 has a close relationship with AJ. However, the ECSM2 distribution along the cell junction appeared to not completely overlap with the pattern of β-catenin in HUVEC. This raises the possibility that ECSM2 might belong to a non-AJ junction type. It is known that multiple adhesion systems may coexist in the same cell [35], [36]. Conceptually, different CAMs could coassemble into the same junction type, exhibiting an extensive distribution overlap. Alternatively, each adhesion system could develop separately and the components might exhibit a mutually exclusive distribution. Importantly, the junctions are dynamic and specific relationships might change in response to a variety of stimuli [37]. Interestingly, an earlier study reported that platelet endothelial adhesion molecule-1 (PECAM-1 or CD31), a member of the IgSF superfamily, localizes at EC junctions but is not associated with either AJ or TJ [36]. This finding along with ours provides an additional line of evidence to the diversity and complexity of EC junctions. Thus, the exact spatial, temporal and functional interrelationships of ECSM2-mediated EC junctions to AJ, TJ, or PECAM-1-associated junctions and definition of critical domains responsible for homophilic cell-cell adhesion deserve more detailed investigation in order to gain greater insight into ECSM2 signaling.

Cell migration is a highly orchestrated process of leading-edge protrusion, turnover of FAs, generation of tractional forces, and rear retraction, which involves precise regulation of cell-cell adhesion and cell-extracellular matrix (ECM) interaction [38]. The current discovery of junctional localization of ECSM2 and our previous findings of inhibitory effects of ECSM2 overexpression on EGF/EGFR-driven migration in a non-EC cell line [3], prompted us to explore the impact of ECSM2 on bFGF-induced EC migration and the underlying signaling mechanisms given the important roles of bFGF in vascular development and EC functions including migration [9], [10], [11], [12]. We found that, while promoting cell-cell adhesion, ECSM2 inhibited the bFGF-directed cell migration using “gain or loss of function" assays by overexpression or knockdown of ECSM2 in ECs. We further defined that ECSM2 modulates bFGF-directed motility via the FGFR-ERK-FAK pathway. In MS1 and HUVEC cells, either overexpression or knockdown of ECSM2 altered bFGF-induced ERK activation. Concomitantly, serine and tyrosine phosphorylation levels of FAK changed and exhibited positive and negative relationships with pERK, respectively. FAK is a crucial signaling component that regulates cell motility through influencing the cytoskeleton, structures of cell adhesion sites and membrane protrusions in response to a variety of stimuli [39]. Growth factor-induced pERK-dependent pFAK(S910), its relationship to dephosphorylation of FAK at Tyr397, and its role in tumor metastasis have been documented in several non-EC systems [20], [24], [40], [41]. Our additional new data in ECs presented in this study point to an intriguing mechanism that serine phosphorylation of FAK could serve as a “brake" on its tyrosine phosphorylation (activation) under certain circumstances. This is reminiscent of the situation of EGFR that we have been investigating, i.e., pERK-dependent receptor threonine phosphorylation counterbalances its tyrosine phosphorylation [25], [26], [27]. In this regard, activation of ERK signaling and a balance between FAK tyrosine phosphorylation (activation) and pERK-dependent serine phosphorylation of FAK are perhaps the prerequisites for ECSM2 to exert its effects on bFGF-directed EC migration (see model below). Indeed, pharmacological inhibition of the ERK pathway eliminated the differences of bFGF-induced pFAK(S910), pFAK(Y397), and cell migration between MS1/ECSM2-GFP and MS1/GFP cells (Figure 9).

**Figure 10:**
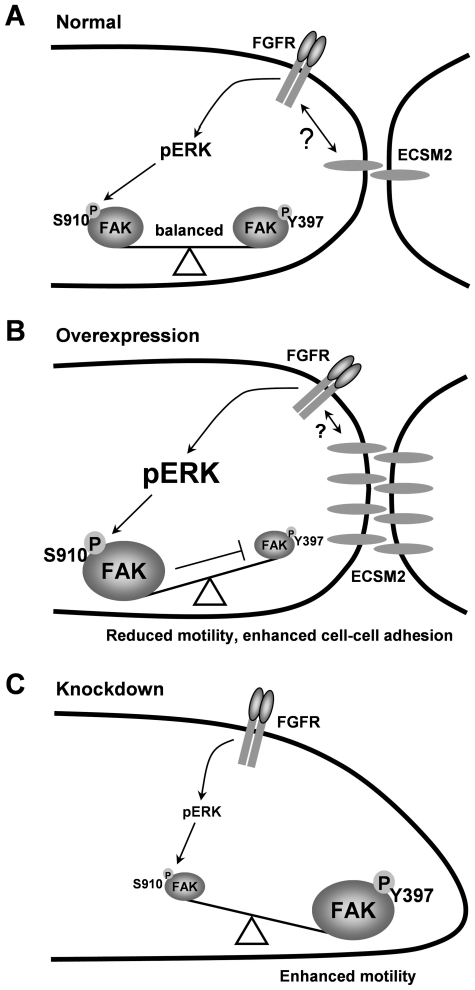
Model of how ECSM2 signals to impact bFGF/FGFR-driven endothelial cell migration. The model is proposed based on the experimental data presented in this study. (A) In ECs, ECSM2 is concentrated at cell-cell contacts and can crosstalk with FGFR (possibly other RTKs) through some unknown interactions (see text for detailed discussion) to activate ERKs (pERK), which causes FAK serine phosphorylation (pS910) to counterbalance FAK tyrosine phosphorylation or activation (pY397). (B) When ECSM2 is overexpressed, cell-cell adhesion and the ECSM2-FGFR crosstalk are enhanced, resulting in elevated pERK, increased pFAK(S910) and decreased pFAK(Y397) levels – pressing the “brake". Thus, bFGF/FGFR-mediated cell migration is inhibited. (C) Knockdown of ECSM2 renders FGFR to signal independently of ECSM2, in turn leading to reduced pERK and decreased (at least not increased) pFAK(S910). Due to the release of the “brake" and subsequent upregulation of pFAK(Y397) – FAK activation, cell motility is enhanced.

RTKs and CAMs are the cell-surface proteins that each can sense the surrounding environment and influence the cell behavior. It is now more believed that a tight relationship exists between RTKs and CAMs, and that they can work together through multiple mechanisms [42]. Based on our experimental data, we propose a working model (Figure 10). Under normal conditions (Figure 10A), ECSM2 can crosstalk with FGFR (perhaps other RTKs) to activate ERKs, which causes FAK phosphorylation at Ser910 to reach a balance between serine and tyrosine phosphorylation (activation) of FAK (the latter could be pre-activated by cell-ECM interaction through integrin signaling [43]). When ECSM2 is overexpressed (Figure 10B), cell-cell contacts and the ECSM2-FGFR crosstalk are enhanced, resulting in elevated ERK activation, increased pFAK(S910) and decreased pFAK(Y397) levels – pressing the “brake". Thus, bFGF-mediated cell migration is inhibited. In contrast, loss of ECSM2 renders FGFR to signal independently of ECSM2, in turn leading to reduced ERK activation and decreased (at least not increased) pFAK(S910). In this case, due to the release of the “brake" and subsequent upregulation of pFAK(Y397), cell motility increases (Figure 10C). Our additional pFAK and pERK data upon EC attachment to fibronectin revealed an inverse relationship between FAK tyrosine phosphorylation (upregulation) and ERK activation (downregulation) (Figure S6), further supporting the idea that alteration in pERK levels can modulate FAK activation via pFAK(S910). This is similar to the case of ECSM2 knockdown (Figure 8), in which downregulated pERK loses its influence over pFAK(S910). Thus, pFAK(S910) remains at the basal level and pFAK(Y397) increases accordingly (no “brake"). Taken together, our results clearly establish a critical role of ECSM2 in bFGF/FGFR-driven cell motility and also imply its connection to integrin signaling through the RTK (FGFR)-ERK-FAK pathway. This undoubtedly introduces a new dimension to controlling the RTK and/or integrin signaling.

We note that the limited literature on ECSM2 has already shown some discrepancy, especially regarding its precise roles in cell migration [3], [4], [6] (and this study) and its anti- or pro-angiogenic effects [4], [5], [6]. As for the impact of ECSM2 knockdown in HUVEC on growth factor-mediated cell migration per se, experimental data from us and three other groups [4], [5], [6] are not consistent. This may have resulted from different assay conditions and individual growth factors that have been used. Armstrong et al. [4] used 20% serum plus 50 µg/ml endothelial cell growth supplement (ECGS) (presumably the same as in full growth medium for HUVEC) and Verma et al. [5] used 10% serum or 25 ng/ml VEGF in endothelial basal medium, respectively, as chemoattractants in their transwell assays. Under these experimental conditions, ECSM2 knockdown in HUVEC resulted in reduced migration. In contrast, we used 10 ng/ml bFGF in serum-free and ECSG-free starvation medium and detected enhanced motility in ECSM2 knockdown cells. Surprisingly, Ikeda et al. did not observe any effect of ECSM2 knockdown on cell migration when 50 ng/ml VEGF was used as a chemoattractant without description of medium, serum and ECGS [6]. It is known that both serum and ECGS contain a variety of growth factors that are required for cell growth. Thus, they could override a specific effect exerted by a particular factor, which is supported by an early finding that ectopic expression of a metastasis suppressor, CD82/KAI-1, in epithelial cells inhibited cell migration only when cells were stimulated with EGF but not with serum [44]. Moreover, although both bFGF and VEGF are considered as potent angiogenic factors capable of inducing EC proliferation, migration, and angiogenesis via interactions with their respective receptors, a number of studies have suggested that they can utilize different signaling pathways (e.g. distinct integrins and upstream/downstream effectors of FAK) to mediate these angiogenic activity [28], [45], [46], [47], [48], [49]. This, at least in part, explains why ECSM2 knockdown has differential roles in modulating VEGF- and bFGF-directed migration. Given that the existing literature on the impact of ECSM2 knockdown on VEGF-stimulated EC proliferation, migration, and Matrigel-based tube formation (in vitro angiogenesis) has been somewhat controversial (pro-angiogenic versus anti-angiogenic) [4], [5], [6], [8], a better understanding of the precise roles of ECSM2 on individual growth factor-mediated angiogenic processes is needed.

Finally, it is important to point out how ECSM2 crosstalks or interacts with the RTKs remains a key question to be addressed, although the impact of ECSM2 on RTK signaling elicited by EGF [3], VEGF [5], and bFGF (this study) is obvious. This could be rather complicated and may not be explained by a simple, straightforward mechanism at this point given the current controversial data on ECSM2 functions related to different growth factors and RTKs as just described. A wealth of evidence [42], stemming from the initial observations that bFGF can only activate FGFRs in the presence of additional molecules [50], [51], has dramatically changed our perception of ligand-dependent activation of RTKs. In fact, CAMs can act as coreceptors for RTKs and the collaboration with CAMs contributes to broaden the diversity of the responses that are achieved by the receptors [42]. For example, localization of E-cadherin at cell-cell junctions or overexpression of E-cadherin blocks the FGF-induced coendocytosis of FGFR1 and E-cadherin, and thus attenuates FGF/FGFR1 signaling to the MEK/ERK pathway in epithelial cells [52]. In contrast, the association of N-cadherin with FGFR1 prevents internalization and downregulation of the receptor and leads to a prolonged ERK signaling [53]. In ECs, VE-cadherin (vascular endothelial cadherin, a unique endothelial AJ component [54], [55]) is associated with VEGFR2 and this association inhibits VEGFR2 phosphorylation and downstream signaling including the ERK pathway [56], [57]. At first glance, these results appear to contradict one another, but in turn mechanistically suggest that the fine-tuning of RTK activation and signaling might be dependent on the presence of specific CAMs (possibly associated with the RTKs) and perhaps the cellular context as well [42]. Nevertheless, these studies could offer several potential mechanisms underlying the crosstalk between ECSM2 and RTKs, which are worthy of future investigation. However, unlike other known CAMs, the lack of predicted functional domains within the intracellular segment of ECSM2 [3], [4] suggests that this could be a new challenge.

In conclusion, the current study is to our knowledge the first demonstration that ECSM2 is an EC junctional component. In concert with its stimulatory role on cell-cell adhesion, ECSM2 attenuates bFGF/FGFR-driven cell migration mechanistically through the ERK-FAK pathway. Our work provides new insights into understanding the roles of ECSM2 in endothelial cell-cell interaction and motility. Restricted expression of ECSM2 to ECs, its concentrated distribution at cell-cell contacts, and close connection with RTKs, β-catenin, ERKs, and FAK raise the possibility that ECSM2 could be a key player in coordinating RTK-, integrin-, and EC junctional component-mediated signaling. Since EC junctions control many key endothelial functions both under normal (quiescent) conditions and in activated situations such as inflammation and angiogenesis [54], [58], our strategic findings also allow speculation that ECSM2 may play an important role in regulation of vascular permeability and stability in diseases. Undoubtedly, this will be an interesting area for future studies.

## Materials and Methods

### Cells, antibodies and reagents

HEK293 cells and mouse endothelial MS1 cells (both from ATCC, Manassas, VA) were grown in DMEM containing 10% fetal bovine serum (FBS) (Mediatech, Manassas, VA). Human umbilical vein endothelial cells (HUVEC) and human dermal microvascular endothelial cells (HDMVEC) (both from Vec Technologies, Rensselaer, NY) were maintained in MCDB-131 complete medium containing 10% FBS and 200 mg/L of endothelial cell growth supplement (ECGS) (Vec Technologies). Human prostate cancer DU145 and breast cancer MCF-7 cells (both from ATCC) were grown in RPMI 1640 medium containing 10% FBS. All antibodies used are detailed in Table S1. Recombinant human bFGF was purchased from Sigma (St. Louis, MO) and the MEK inhibitor PD98059 was from Cell Signaling (Beverly, MA).

### RNA extraction, RT-PCR, and quantitative PCR

Total RNAs were extracted from tissue and cells using TRIzol Reagent (Invitrogen, Carlsbad, CA). cDNA synthesis was performed with SuperScript III First-strand Synthesis Supermix (Invitrogen) and PCR was carried out using Pfu Ultra DNA polymerase (Agilent Technologies, Santa Clara, CA). Quantitative PCR was performed using Platinum SYBR Green qPCR Supermix UDG Kit (Invitrogen) on the iQ5 Real-Time PCR Detection System (Bio-Rad, Hercules, CA). Expression of ECSM2 was normalized to GAPDH.

### Plasmid constructs, cell transfection, and generation of stable cell lines

cDNAs encoding full-length human and mouse ECSM2, respectively, were obtained by RT-PCR and cloned into pEGFP-N1 (Clontech Laboratories, Palo Alto, CA) or p3×FLAG-CMV-14 (Sigma), as described previously [3], [16]. Plasmid DNAs were transfected into HEK293 cells with Lipofectamine 2000 (Invitrogen) and stable cell lines were generated by selection with 1 mg/ml of G418 [3], [16]. The MS1 cells stably expressing ECSM2-GFP or GFP alone were obtained by cotransfection of respective expression construct with pcDNA3.1-Hygro (Invitrogen) and selected with 0.2 mg/ml of hygromycin.

### Generation and screening of rabbit anti-ECSM2 monoclonal antibody (RabMAb)

The cDNA fragment encoding the entire intracellular domain (ICD) of human ECSM2 was cloned into pGEX-4T-1 (GE Healthcare, Piscataway, NJ) or pET-16b (EMD Chemicals, Gibbstown, NJ). The two recombinant plasmids were used to transform *E. coli* BL21(DE3)pLys strain and expression of glutathione *S*-transferase (GST)-tagged or His-tagged ICD fusion proteins in *E. coli* was induced with IPTG. The GST- and His-fusion proteins were purified using glutathione-Agarose 4B (USB Corporation, Cleveland, OH) and Ni-NTA Agarose (Qiagen, Valencia, CA), respectively. The purified GST-ICD fusion proteins were used as the antigen to generate rabbit monoclonal antibody (RabMAb) using the custom antibody services provided by Epitomics, Inc (Burlingame, CA). Briefly, two New Zealand white rabbits were injected with GST-ICD proteins (0.2 mg proteins per injection, 4–5 injections). Two test bleeds were collected and used for primary screening sequentially by ELISA against His and/or GST and by immunoblotting using purified His-ICD proteins and total protein extracts from HEK293 cells overexpressing ECSM2-GFP versus GFP control. To generate hybridomas, isolated spleen cells from one immunized rabbit (positive in antiserum screening) were fused with rabbit fusion partner cells (plasmacytoma) [59]. Positive hybridoma clones were identified by differential ELISA and further screened by immunoblotting in a panel of cell lines including HEK293 cells overexpressing ECSM2-GFP and endothelial cell lines endogenously expressing ECSM2 (Figure 1).

### Knockdown of ECSM2 in HUVEC cells

Predesigned siRNAs specifically targeting human ECSM2 gene (Sequence no. 146: 5′-GAGUUCAGGGCUGUCUGAA-3′ (sense), 5′-UUCAGACAGCCCUGAACUC-3′ (antisense); Sequence no. 147: 5′-CAUGAAUAAUGGCAAACAA-3′ (sense), 5′-UUGUUUGCCAUUAUUCAUG-3′ (antisense)) and universal negative (nonspecific) control siRNA were purchased from Sigma. HUVECs were transfected with 200 nM of siRNA duplexes using HiPerFect Transfection Reagent (Qiagen), as described previously [26]. The transfectants were used for various assays 48–72 h post transfection as specified in each experiment.

### Immunofluorescent staining and microscopy

Cells were grown on glass coverslips precoated with gelatin (2%) or fibronectin (20 µg/ml) and fixed in 4% paraformaldehyde for 15 min, permeabilized with 0.25% Triton X-100 in PBS containing 1% BSA for 15 min, stained with mouse anti-FAK mAb clone 4.47 (Millipore), rabbit anti-ECSM2 RabMAb 71-1 (hybridoma supernatant), and/or mouse anti-β-catenin mAb (BD Biosciences) as specified in each experiment. The mouse and rabbit antibodies were detected with FITC- or TRITC-conjugated anti-mouse or anti-rabbit IgG antibody as specified in each experiment. The coverslips were mounted onto microscope slides in Vectashield mounting medium for fluorescence containing DAPI (Vector Laboratories, Burlingame, CA) and fluorescent images were visualized and captured using a Zeiss Axio Imager upright fluorescent microscope and a Zeiss Laser Scanning Confocal Microscope LSM 710 (Carl Zeiss USA, Thornwood, NY), as described previously [3], [16], [26].

### Cell aggregation assay

Cell aggregation assays were performed using a modified protocol as described elsewhere [60]. Briefly, cells were washed with PBS (without Ca^2+^ and Mg^2+^), trypsinized with 0.05% trypsin containing 0.5 mM Ca^2+^, and washed with HCMF buffer (10 mM HEPES, pH 7.4, 137 mM NaCl, 5.4 mM KCl, 0.34 mM Na_2_HPO_4_·12H_2_O, and 0.1% glucose) to remove the Ca^2+^. The cells were resuspended at 10^5^ per 0.5 ml in HCMF containing 1 mM Ca^2+^ in 1.5 ml tubes and rotated at 37°C for 30 min. The cells were then transferred to a 24-well plate and shaken for additional 30 min. Images of 8 random areas per well were captured using a Zeiss Axiovert 200 M inverted microscope (Carl Zeiss USA) to observe cell aggregates. Approximately 1,000 cells were counted under each condition. The aggregation index (*N_t_/N_0_*) was calculated (*N_t_* = total particle number, including aggregates and single cells; *N_0_* = total cell number in cell suspension).

### Transwell assay

BD Falcon 8- µm pore inserts (BD Biosciences, San Diego, CA) were placed in a 12-well plate containing 1 ml of starvation medium with or without bFGF (10 ng/ml) (lower chamber). Serum-starved cells were trypsinized and counted. Cell suspensions in 0.5 ml of starvation medium were then added to the transwell inserts (1×10^5^ cells per well) (upper chamber) and incubated at 37°C for 5 h. In the case of PD98059 pretreatment, serum-starved cells were pretreated with PD98059 (50 µM) for 1 h prior to trypsinization and PD98059 (50 µM) was added to the media in both lower and upper chambers. At the end of incubation, the cells were fixed by submerging the transwell inserts in 4% paraformaldehyde and counterstained with DAPI (0.5 µg/ml). The non-migrated cells on the top side of the membrane were removed with wet cotton swabs. Air dried membranes were cut out from the transwell inserts, mounted onto microscope slides, and examined using a Zeiss Axio Imager upright fluorescent microscope (Carl Zeiss USA), as described previously [26]. The cells from 12 randomly selected fields were counted.

### Cell starvation, inhibitor treatment, stimulation, protein extraction, and immunoblotting

Cell starvation, drug pretreatment, stimulation, protein extraction, and immunoblotting (IB) were performed as previously described [13], [25], [26], [27]. Briefly, cells were starved in serum-free (and ECGS-free, if applicable) medium containing 0.5% BSA for 5-16 h, pretreated with PD98059 (50 µM) or vehicle (DMSO) for 1 h, and then stimulated with bFGF (10 ng/ml) at 37°C for 15 min. The cells were harvested, lysed, and solubilized in Lysis Buffer A (50 mM Tris-HCl (pH8.0), 2 mM EDTA, 150 mM NaCl, 100 mM NaF, 10% glycerol, and 1% SDS) with protease and phosphatase inhibitors. The cell extracts were quantitated with bicinchoninic acid (BCA) reagents (Pierce, Rockford, IL) and used for immunoblotting with antibodies as specified in each experiment. Immunoblotting signals were detected with SuperSignal chemiluminescent substrate (Pierce) and images were captured using a Kodak 4000 MM molecular imager.

### Immunoprecipitation and enzymatic deglycosylation

Enzymatic deglycosylation assays were performed using either immunoprecipitated proteins or cell lysates. For HUVEC, immunoprecipitation (IP) experiments were performed as we described elsewhere [25], [27]. Specifically, HUVEC cells were lysed in Lysis Buffer B (50 mM Tris-HCl (pH 8.0), 2 mM EDTA, 150 mM NaCl, 100 mM NaF, 10% glycerol, and 1% Triton X-100) with protease and phosphatase inhibitors. Cell extracts (1.5 mg) were mixed with 150 µl of anti-ECSM2 RabMAb 71-1 (hybridoma supernatant) and incubated at 4°C overnight with continuous agitation. Protein A-Sepharose beads (Amershan Pharmacia Biotechnology, Arlington Heights, IL) were added and incubated at 4°C for additional 2 h. The beads were washed three times with Lysis Buffer B adjusted to 0.5% Triton X-100. Precipitated proteins were eluted by boiling the beads in 1×Glycoprotein Denaturing Buffer (0.5% SDS and 40 mM DTT) for 10 min. Enzymatic deglycosylation reaction (total volume of 50 µl) was accomplished by adjusting the buffer to 50 mM sodium phosphate (pH 7.5) and 1% Nonidet P-40 and adding 5 µl of Deglycosylation Enzyme Mix consisting of PNGase F, Endo-α-N-Acetylgalactosaminidase, Neuraminidase, β1-4 Galactosidase, and β-N-Acetylglucosaminidase (New England BioLabs, Beverly, MA) and incubated at 37°C for 4 h, according to the manufacturer's suggestions. Nondeglycosylated controls were subjected to the same treatment, but without the addition of the Deglycosylation Enzyme Mix, as previously described [13].

For HEK293 cells stably expressing ECSM2-FLAG, cells were lysed in RIPA buffer (25 mM Tris-HCl (pH 7.6), 150 mM NaCl, 1% Nonidet P-40, 1% sodium deoxycholate, and 0.1% SDS) with protease and phosphatase inhibitors. Cell lysates (100 µg) were denatured by boiling in 1×Glycoprotein Denaturing Buffer (0.5% SDS and 40 mM DTT) for 10 min and used directly in enzymatic deglycosylation reactions as above. After glycosidase digestion, SDS-PAGE sample buffer was added and incubated at 100°C for 5 min. The samples were analyzed by immunoblotting.

### Densitometry and statistical analysis

Densitometric quantification of digital immunoblotting images was performed using Kodak Molecular Imaging Software (Version 4.0) [3], [26]. All statistical data were from multiple experiments or measurements and presented as mean±SEM. The significance (*p* value) of differences was estimated using unpaired Student's *t*-test and *p*<0.05 was considered significant.

## Supporting Information

Figure S1 **Initial identification of concentrated distribution of ECSM2 at cell-cell contacts.** (A and B) Generation of MS1 cells stably expressing ECSM2-GFP or GFP alone, verified by immunoblotting with anti-GFP (A) and by visualization of GFP fluorescence (B). The filopodia-like structures (B) are indicated by *arrowheads*. Scale bar, 20 µm. (C and D) Localization of ECSM2 (tagged with GFP or FLAG) at cell-cell junctions when heterologously expressed in MS1 (C) and HEK293 (D) cells. Merged images of GFP (*green*) or FLAG (*red*) with DAPI (*blue*) are shown. Scale bar, 20 µm.
(TIF)

Figure S2 **ECSM2 does not localize to focal adhesions (FAs).** MS1 cells expressing ECSM2-GFP were costained with anti-FAK antibody and DAPI. ECSM2-GFP (*green*), FAK (*red*), DAPI (*blue*) staining, and merged image are shown. Concentrated distribution of ECSM2-GFP at cell-cell contacts are indicated by *arrows*. Scale bar, 20 µm.
(TIF)

Figure S3 **bFGF-induced ERK signaling is more robust than VEGF in ECs.** (A) Serum-starved MS1 cells were stimulated with 10 ng/ml of VEGF or bFGF for 15 min. Protein extracts were analyzed by immunoblotting with anti-pERK and anti-total ERK antibodies, respectively. (B) HUVEC cells were starved in the media free of serum and endothelial cell growth supplements (ECGS) for 8 h, and then stimulated with 10, 25, and 50 ng/ml of VEGF or bFGF for 15 min. Protein extracts were analyzed by immunoblotting with anti-pERK and anti-total ERK antibodies, respectively.
(TIF)

Figure S4 **ECSM2 overexpression attenuates bFGF-induced FAK phosphorylation at Tyr576 and Tyr577.** (A) Serum-starved GFP- or ECSM2-GFP-overexpressing MS1 cells were stimulated with vehicle (-) or bFGF (10 ng/ml) for 15 min. Protein extracts were analyzed by immunoblotting with anti-pFAK(Y576/Y577) and anti-total FAK, respectively. (B) Statistical analysis (densitometry) of pooled data of pFAK(Y576/Y577) from four independent experiments. Data are mean±SEM. **, *P*<0.01.
(TIF)

Figure S5 **The pFAK(S910) level is not altered by ECSM2 knockdown.** HUVECs were transfected with nonspecific (NS) or ECSM2 siRNAs for 72 h, starved for 8 h, and then stimulated with vehicle (-) or bFGF (10 ng/ml) for 15 min. Protein extracts were analyzed by immunoblotting with anti-pFAK(S910) as shown in Fig. 9A. Statistical analysis (densitometry) of pooled data of pFAK(S910) from three independent experiments did not show statistical significance. Data are mean±SEM.
(TIF)

Figure S6 **There exists a balance among ERK activation and FAK tyrosine and serine phosphorylation upon cell attachment to fibronectin.** HUVECs were trypsinized, resuspended in complete growth media, seeded in fibronectin-precoated 35 mm dishes, and cell attachment was allowed for 0, 20, 40 and 60 min. Protein extracts were analyzed by immunoblotting with anti-pFAK(Y397), anti-pFAK(Y576/Y577), anti-pFAK(S910), anti-total FAK, anti-pERK, anti-total ERK, or anti-β-actin antibodies, as indicated.
(TIF)

Table S1 **List of antibodies used in the study.**
(DOC)
