# Supplementary figures and images for: Endothelial Cell-Specific Molecule 2 (ECSM2) Localizes to Cell-Cell Junctions and Modulates bFGF-Directed Cell Migration via the ERK-FAK Pathway
Source: PLoS One. 2011 Jun 24;6(6):e21482. doi: 10.1371/journal.pone.0021482 (PMC3123356; doi:10.1371/journal.pone.0021482)

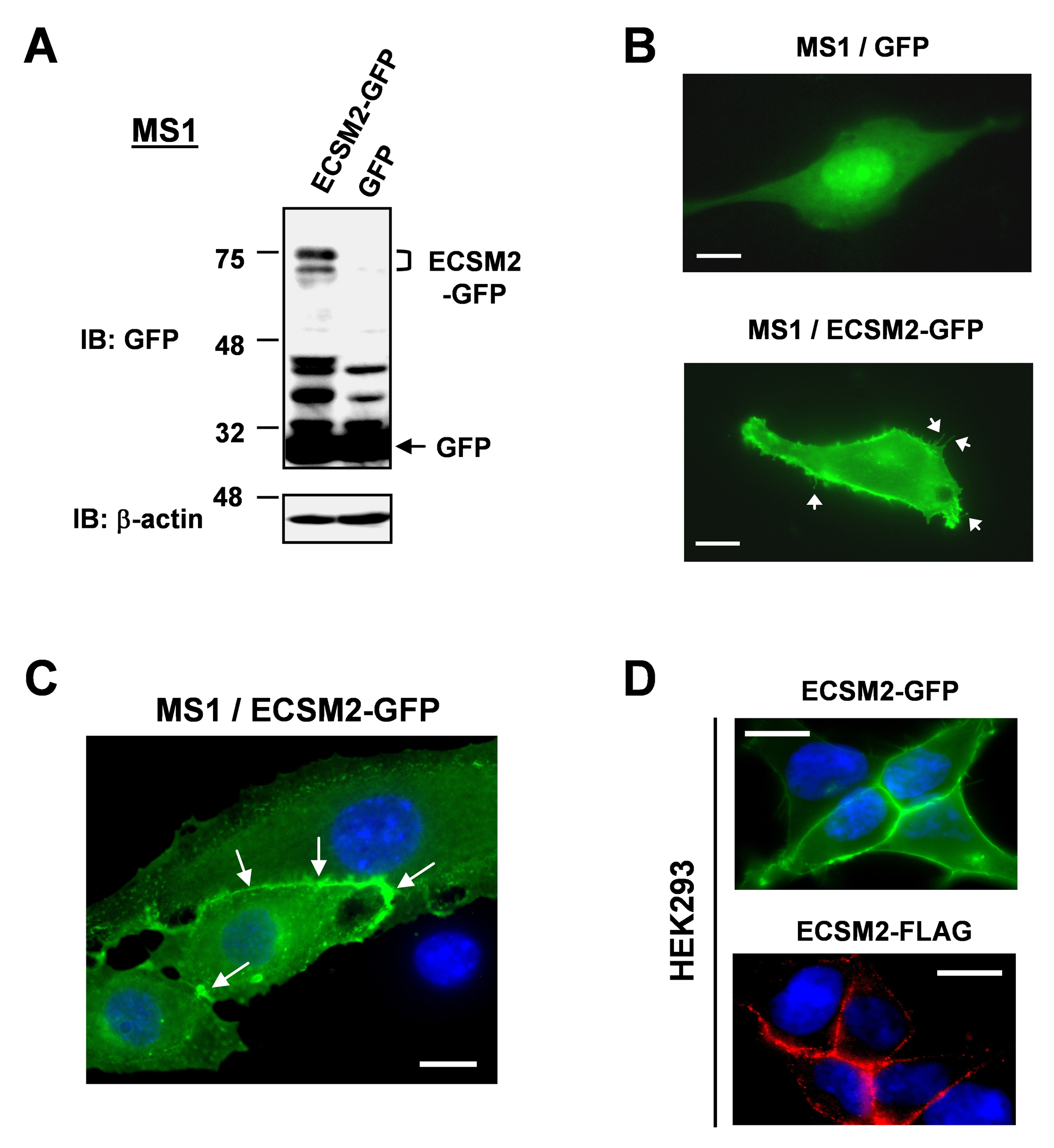

Supplement: Figure S1 — Initial identification of concentrated distribution of ECSM2 at cell-cell contacts. (A and B) Generation of MS1 cells stably expressing ECSM2-GFP or GFP alone, verified by immunoblotting with anti-GFP (A) and by visualization of GFP fluorescence (B). The filopodia-like structures (B) are indicated by arrowheads. Scale bar, 20 µm. (C and D) Localization of ECSM2 (tagged with GFP or FLAG) at cell-cell junctions when heterologously expressed in MS1 (C) and HEK293 (D) cells. Merged images of GFP (green) or FLAG (red) with DAPI (blue) are shown. Scale bar, 20 µm. (TIF) [file pone.0021482.s001.tif]

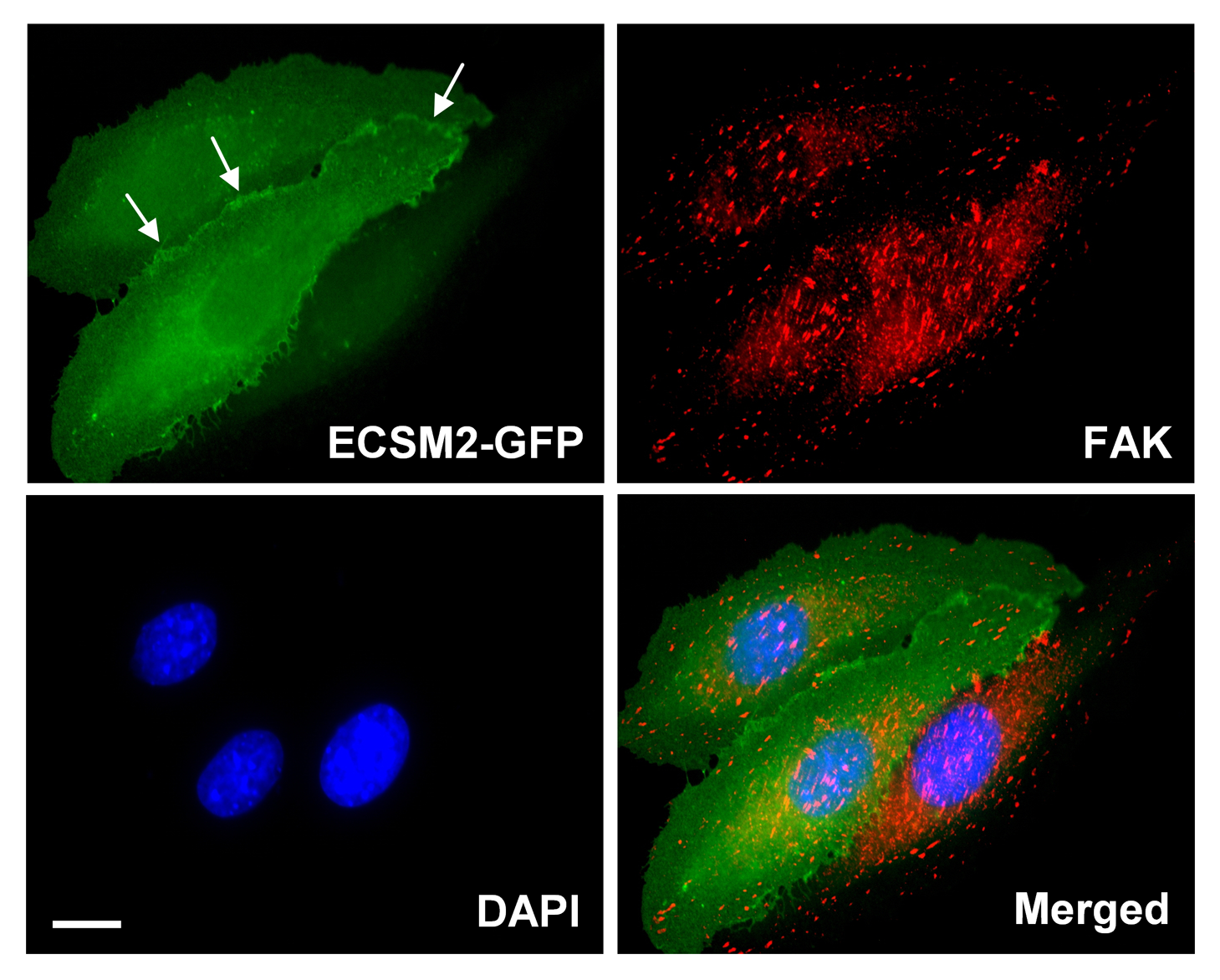

Supplement: Figure S2 — ECSM2 does not localize to focal adhesions (FAs). MS1 cells expressing ECSM2-GFP were costained with anti-FAK antibody and DAPI. ECSM2-GFP (green), FAK (red), DAPI (blue) staining, and merged image are shown. Concentrated distribution of ECSM2-GFP at cell-cell contacts are indicated by arrows. Scale bar, 20 µm. (TIF) [file pone.0021482.s002.tif]

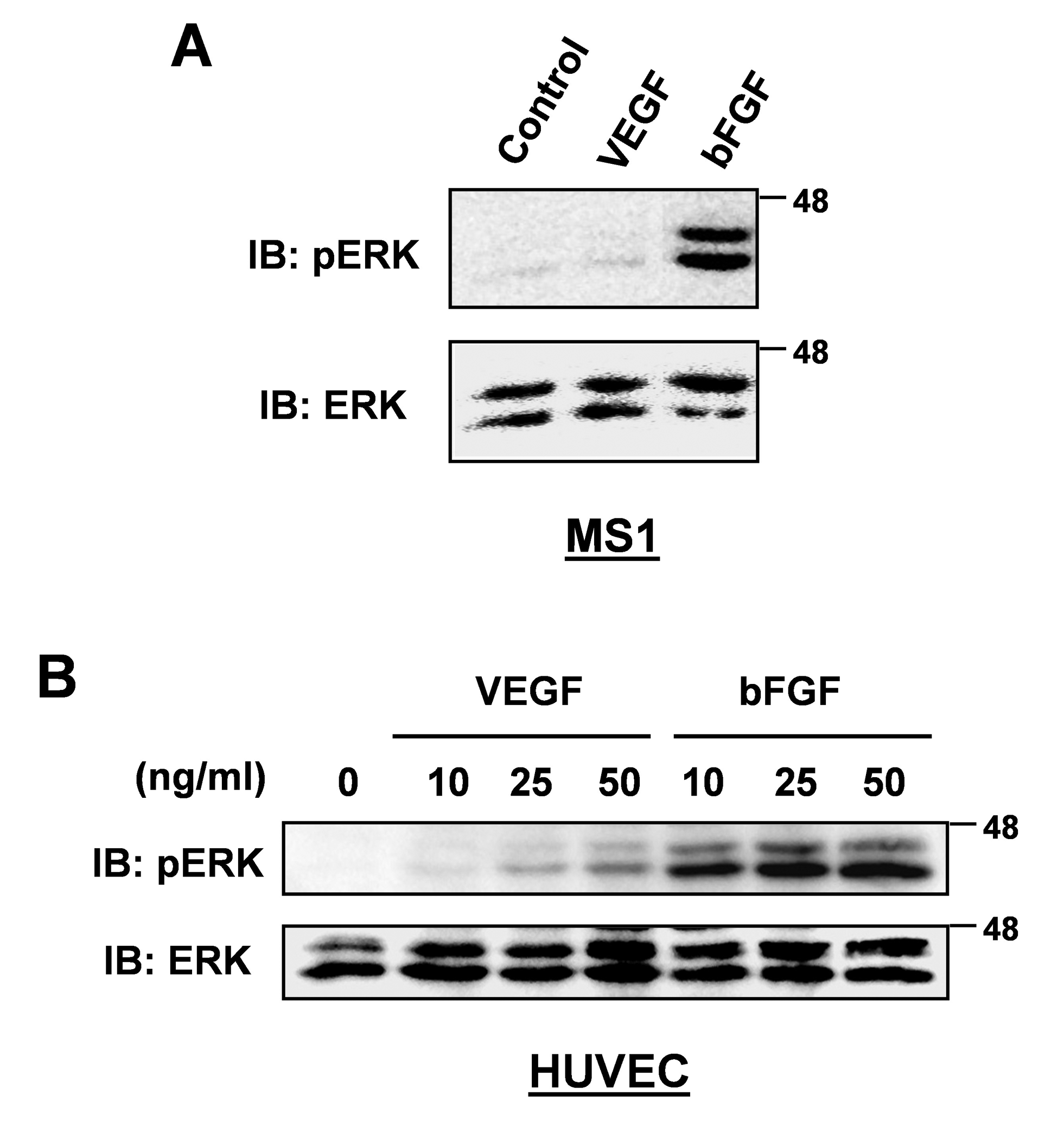

Supplement: Figure S3 — bFGF-induced ERK signaling is more robust than VEGF in ECs. (A) Serum-starved MS1 cells were stimulated with 10 ng/ml of VEGF or bFGF for 15 min. Protein extracts were analyzed by immunoblotting with anti-pERK and anti-total ERK antibodies, respectively. (B) HUVEC cells were starved in the media free of serum and endothelial cell growth supplements (ECGS) for 8 h, and then stimulated with 10, 25, and 50 ng/ml of VEGF or bFGF for 15 min. Protein extracts were analyzed by immunoblotting with anti-pERK and anti-total ERK antibodies, respectively. (TIF) [file pone.0021482.s003.tif]

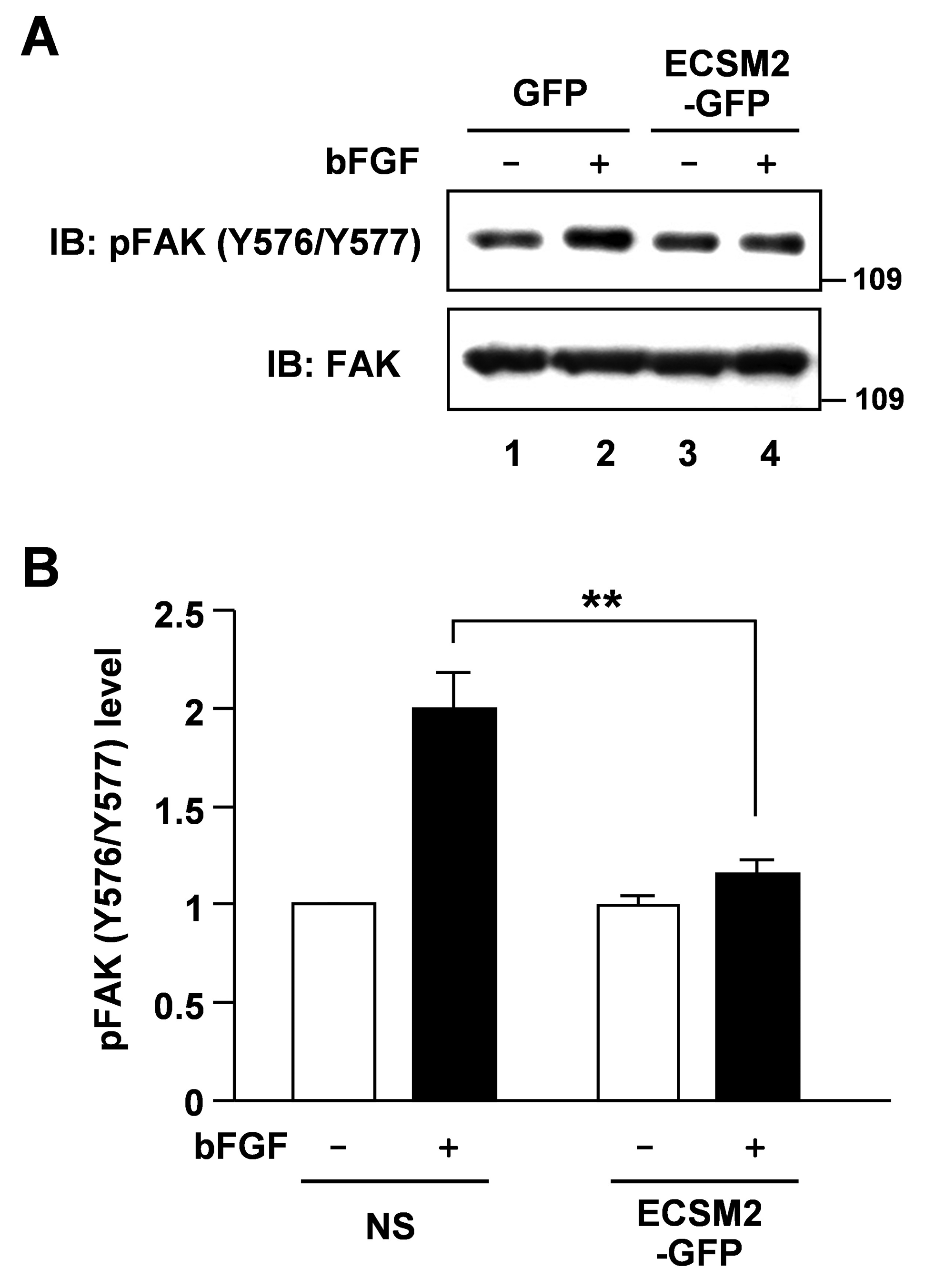

Supplement: Figure S4 — ECSM2 overexpression attenuates bFGF-induced FAK phosphorylation at Tyr576 and Tyr577. (A) Serum-starved GFP- or ECSM2-GFP-overexpressing MS1 cells were stimulated with vehicle (-) or bFGF (10 ng/ml) for 15 min. Protein extracts were analyzed by immunoblotting with anti-pFAK(Y576/Y577) and anti-total FAK, respectively. (B) Statistical analysis (densitometry) of pooled data of pFAK(Y576/Y577) from four independent experiments. Data are mean±SEM. **, P<0.01. (TIF) [file pone.0021482.s004.tif]

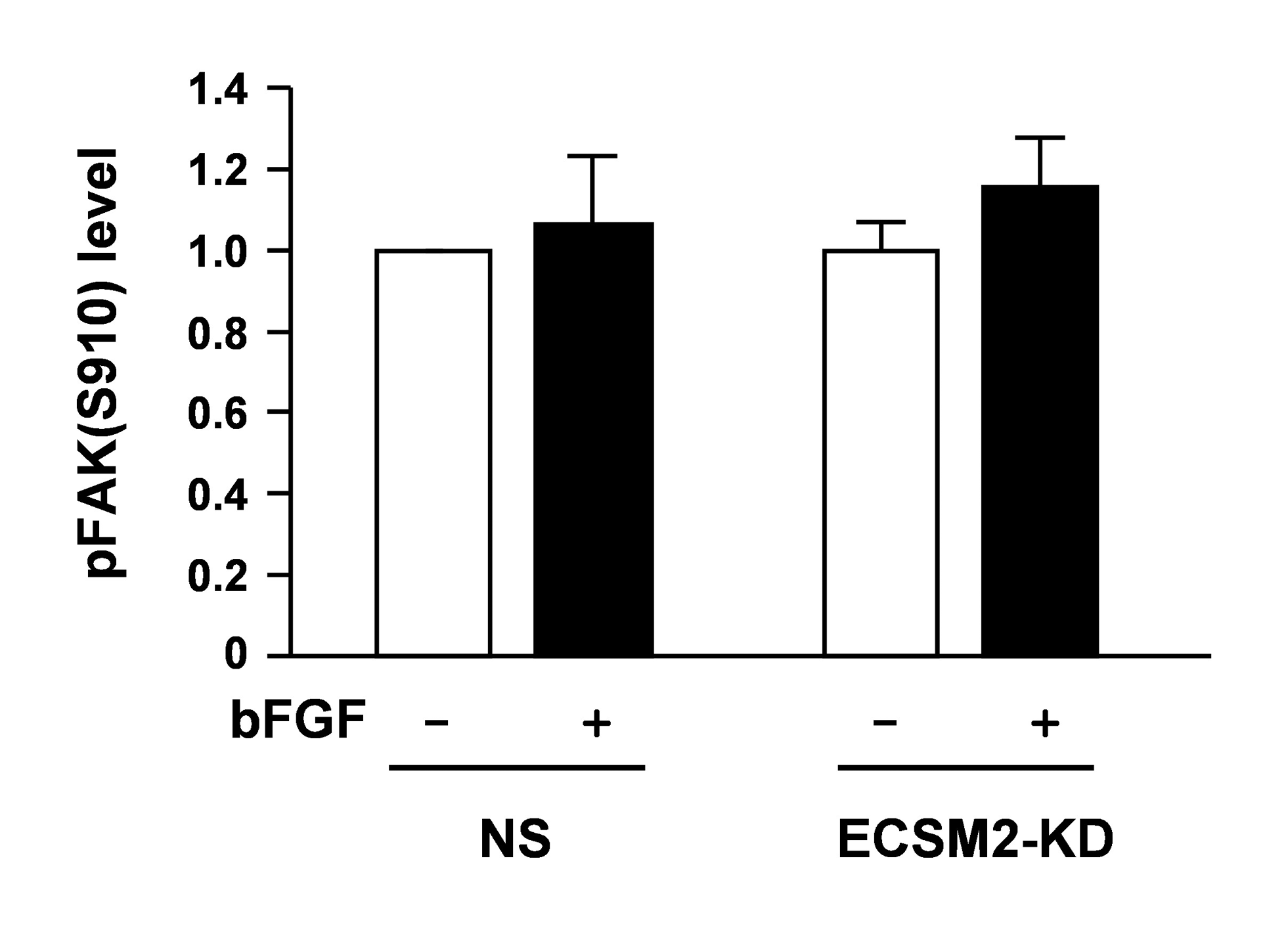

Supplement: Figure S5 — The pFAK(S910) level is not altered by ECSM2 knockdown. HUVECs were transfected with nonspecific (NS) or ECSM2 siRNAs for 72 h, starved for 8 h, and then stimulated with vehicle (-) or bFGF (10 ng/ml) for 15 min. Protein extracts were analyzed by immunoblotting with anti-pFAK(S910) as shown in Fig. 9A. Statistical analysis (densitometry) of pooled data of pFAK(S910) from three independent experiments did not show statistical significance. Data are mean±SEM. (TIF) [file pone.0021482.s005.tif]

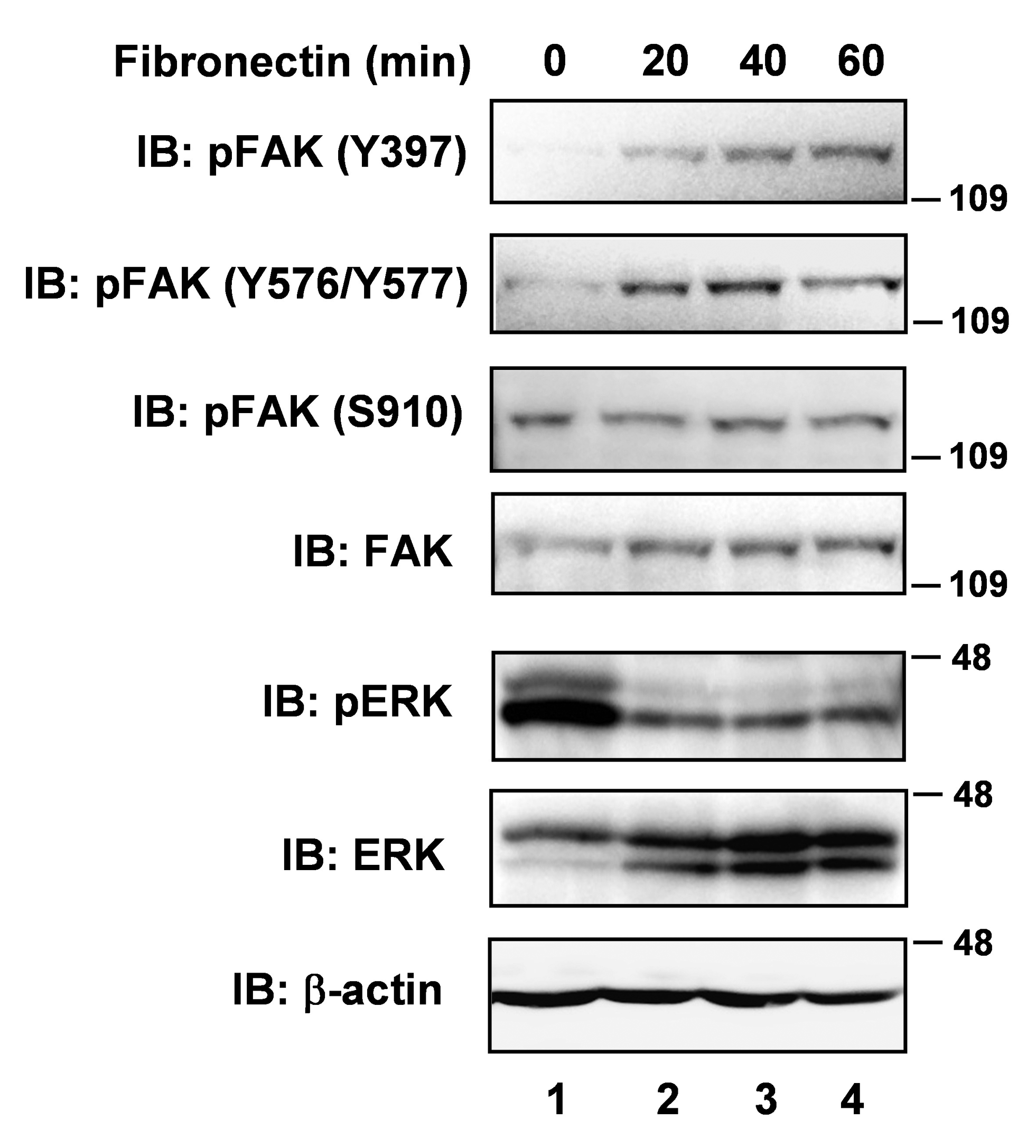

Supplement: Figure S6 — There exists a balance among ERK activation and FAK tyrosine and serine phosphorylation upon cell attachment to fibronectin. HUVECs were trypsinized, resuspended in complete growth media, seeded in fibronectin-precoated 35 mm dishes, and cell attachment was allowed for 0, 20, 40 and 60 min. Protein extracts were analyzed by immunoblotting with anti-pFAK(Y397), anti-pFAK(Y576/Y577), anti-pFAK(S910), anti-total FAK, anti-pERK, anti-total ERK, or anti-β-actin antibodies, as indicated. (TIF) [file pone.0021482.s006.tif]
