# Supplementary material for: Endothelial Cell-Specific Molecule 2 (ECSM2) Localizes to Cell-Cell Junctions and Modulates bFGF-Directed Cell Migration via the ERK-FAK Pathway
Source: PLoS One. 2011 Jun 24;6(6):e21482. doi: 10.1371/journal.pone.0021482 (PMC3123356; doi:10.1371/journal.pone.0021482)
Supplement: Table S1 — List of antibodies used in the study. (DOC) [file pone.0021482.s007.doc]

**Supplemental Data**

**Table S1. List of antibodies used in the study.**

| **Description of Antibody** | **Name and Address of Vendor** | **Catalogue No.** |
| --- | --- | --- |
| HisProbe-HRP | Pierce, Rockford, IL | 15165 |
| GFP | Sigma, St. Louis, MO | G1544 |
| FLAG M2 | Sigma, St. Louis, MO | F1804 |
| ERK1/2 | Millipore (Upstate), Temecula, CA | 06-182 |
| pERK1/2 (active MAPK) | Promega, Madison, WI | V8031 |
| FAK, rabbit polyclonal | Cell Signaling, Beverly, MA | 3285 |
| FAK, rabbit monoclonal | Epitomics, Burlingame, CA | 1700-1 |
| FAK mAb (clone 4.47) | Millipore (Upstate), Temecula, CA | 05-537 |
| pFAK(Y397) | Cell Signaling, Beverly, MA | 3283 |
| pFAK(Y576/Y577) | Cell Signaling, Beverly, MA | 3281 |
| pFAK(S910) | Invitrogen, Carlsbad, CA | 44-596G |
| β-actin mAb | Sigma, St. Louis, MO | A5441 |
| β-catenin mAb | BD Biosciences (Pharmingen), San Diego, CA | 610153 |
| Fluorescein isothiocyanate (FITC)-conjugated goat anti-mouse IgG Ab | Jackson ImmunoResearch Laboratories, West Grove, PA | 115-095-146 |
| Tetramethyl Rhodamine Isothiocyanate (TRITC) -conjugated goat anti-mouse IgG Ab | Jackson ImmunoResearch Laboratories, West Grove, PA | 115-025-146 |
| Tetramethyl Rhodamine Isothiocyanate (TRITC)-conjugated goat anti-rabbit IgG Ab | Jackson ImmunoResearch Laboratories, West Grove, PA | 111-025-144 |
| Goat anti-rabbit secondary Ab | Pierce, Rockford, IL | 31460 |
| Goat anti-mouse secondary Ab | Pierce, Rockford, IL | 31430 |
